# The early-life fecal microbiota is associated with litter of origin but not with susceptibility to ETEC F4ab-mediated post-weaning diarrhea in CHCF1 genotyped pigs

**DOI:** 10.1371/journal.pone.0323875

**Published:** 2025-05-29

**Authors:** Martin Peter Rydal, Louise Ladefoged Poulsen, Jens Peter Nielsen

**Affiliations:** Department of Veterinary and Animal Sciences, Faculty of Health and Medical Sciences, University of Copenhagen, Frederiksberg, Denmark; University of Tripoli, LIBYA

## Abstract

**Introduction:**

Universal biomarkers in the fecal microbiota that predict susceptibility to post-weaning diarrhea (PWD) would be valuable for future intervention strategies. Genetic susceptibility to enterotoxigenic *E. coli* (ETEC) infection in pigs is a major determinant of PWD and may unfavourably alter early-life gut microbiota composition. We investigated whether pigs genetically susceptible to ETEC F4ab/ac had different fecal microbiota composition and diversity pre- and post-weaning compared to genetically resistant pigs.

**Method:**

Fecal microbiotas were characterized using long-read sequencing of the 16S rRNA gene in 24 CHCF1 heterozygous susceptible (RS) and 24 CHCF1 homozygous resistant (RR) pigs. These pigs were tested at early lactation (post-natal day (PND) 8) and late lactation (PND 22), which are critical periods for microbiota development and immune maturation. Twelve pigs from each group were weaned and transported to an experimental facility at PND 23, and were tested again at PND 24, prior to an ETEC F4ab challenge. This enabled studying immediate fecal microbiota changes after weaning and investigating whether CHCF1 RS pigs had compromised microbiotas compared to CHCF1 RR pigs preceding infection.

**Results:**

Across time, CHCF1 RS pigs had a higher number of observed OTUs (coef: 103, 95% CI [18.90; 192.76], p = 0.01) compared to CHCF1 RR pigs. There were no significant differences in the overall bacterial communities or differentially abundant taxa between genotypes. Littermates had bacterial communities more similar to each other compared to non-littermates during lactation (PND 8: R^2^ = 0.2, p = 0.001 and PND 22: R^2^ = 0.23, p = 0.001) and the litter effect persisted after weaning (PND 24: R^2^ = 0.4, p = 0.001).

**Conclusion:**

We did not find major differences in the fecal microbiota between CHCF1 genotypes, pre- or post-weaning, that could help explain subsequent susceptibility to ETEC F4ab-mediated PWD. Litter explained a major part of the variation in the overall fecal bacterial community between pigs in the study.

## Introduction

Post-weaning diarrhea (PWD) caused by enterotoxigenic *E. coli* (ETEC) is a major problem in pig production that is often controlled with antibiotic treatments [1], which lead to antimicrobial resistance [2], a potential threat to porcine and human health [3].

Pre-weaning fecal microbiota composition and diversity have been suggested to influence subsequent vulnerability to PWD [4,5]. Specifically, lower early-life abundance of *Prevotellaceae, Lachnospiraceae, Ruminococaceae* and *Lactobacillaceae* and higher evenness were associated with PWD susceptibility, when comparing PWD affected and healthy pigs [4]. Finding biomarkers in the pre-weaning fecal microbiota that could be used as early predictors for PWD resilience and susceptibility would give scientific grounds for early-life intervention with gut modulating agents and could serve as early warnings for piglets at risk.

Host genotype is a critical factor in determining susceptibility to ETEC fimbriated with F4 [6] and F18 [7], which account for a majority of isolates recovered from PWD outbreaks in the first two weeks after weaning [8,9]. In addition to enabling fimbriae adhesion, FUT1 genotype (determining F18ab/ac adhesion [7]) and MUC4 genotype (associated with F4ab/ac adhesion [10]) may influence the bacterial community and affect gut homeostasis, as shown in relation to immune regulation and intestinal barrier function [11–13]. The genetic marker CHCF1 was found superior to MUC4 as predictor of susceptibility for F4ab/ac adhesion in vitro [14], as well as clinical ETEC F4ab/ac-associated diarrhea in vivo [15,16].

The aim of this study was to investigate if and how the fecal microbiota differed between CHCF1 genotypes. In particular, to investigate if CHCF1 RS pigs had lower diversity and lower abundance of certain commensals (*Prevotellaceae, Lachnospiraceae, Ruminococaceae* and *Lactobacillaceae*) pre and post weaning, which could contribute to explaining the pigs’ subsequent vulnerability to PWD. Additionally, the effect of litter of origin on the fecal microbiota was analyzed pre- and post-weaning.

## Materials and methods

### Ethical statement

The part of the study conducted at the herd was ethically reviewed and approved by the Animal Ethics Institutional Review Board at the Department of Veterinary and Animal Sciences, University of Copenhagen, approval number 2022-11-PNH-017A. The part of the study that was conducted at the experimental facility was approved by the Danish Animal Experiments Inspectorate, license number 2019-15-0201-00166.

### Animals

A total of 48 female, Landrace x Yorkshire x Duroc (LY-D) pigs were included in the study. The pigs were born on the same day and originated from eight litters (5–9 pigs per litter). Only pigs weighing between 1–2 kg at birth were included in the study. The pigs were not cross-fostered and were kept with the mother sow during the entire pre-weaning period. Sow parity ranged from 2–8. Pigs received an intramuscular injection of amoxicillin trihydrate at post-natal day (PND) 1 and were treated against coccidia with Toltrazuril at PND 4. At the herd, pigs had access to milk dispensers with formula milk as supplement to sow milk and creep feed from PND 16. At PND 23, 24 of the pigs were weaned and transported to an experimental facility for an ETEC challenge trial. In the animal facility, the 12 CHCF1 RS and 12 CHCF1 piglets where housed in two separate rooms, with one pen per room to avoid cross contamination. The health status of the pigs was monitored at least 3 times each day prior to inoculation. Pigs were scored clinically by a veterinarian twice daily. In case of pigs meeting humane endpoints [16], they would be sedated followed by euthanasia using pentobarbital by intracardial injection. However, all pigs in the study remained healthy prior to inoculation. Information regarding the experimental facility housing, feed, health monitoring, humane endpoints, and euthanasia has been described in detail for the experimental trial [16].

### Experimental design

**Fig 1:**
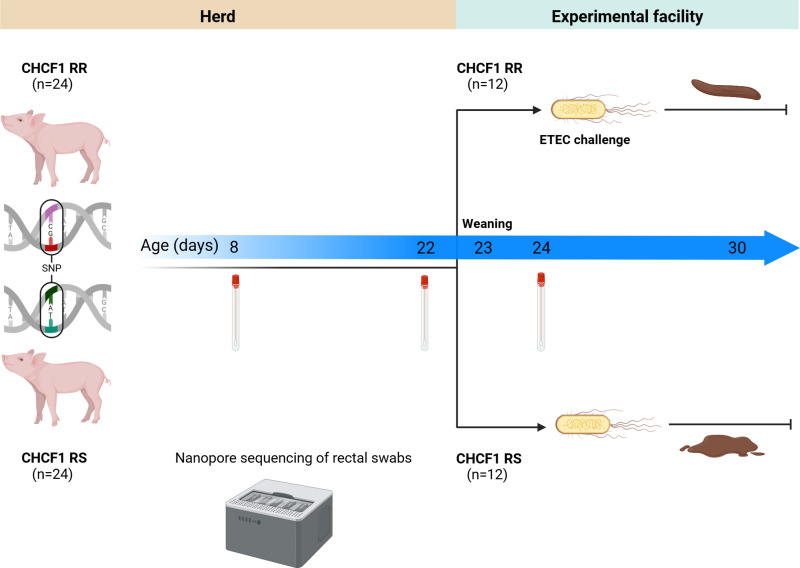
Study timeline. Twenty-four CHCF1 homozygous resistant (RR) pigs and 24 CHCF1 heterozygous susceptible (RS) pigs had swabs taken at post-natal day (PND) 8 and 22 for pre-weaning comparison of their fecal microbiota using nanopore long-read sequencing of the 16S rRNA gene. Twelve pigs in each group were weaned at PND 23 and transported to an experimental facility. After one day of acclimatization (PND 24), swabs for fecal microbiota comparison were collected, after which pigs received an enterotoxigenic *E. coli* (ETEC) F4 challenge. Created with BioRender.com.

The study was a longitudinal, observational study with stratified randomization. The study population of 48 pigs from the same eight sows was randomized in blocks by CHCF1 genotype to achieve 24 CHCF1 heterozygous susceptible (RS) and 24 homozygous resistant (RR) pigs. The randomization process is illustrated in S1 Fig. The fecal microbiotas of the 24 CHCF1 RS and 24 CHCF1 RR pigs were compared at early lactation (PND 8) and at late lactation (PND 22). Fecal swabs were also collected for microbiota sequencing from the eight sow mothers at PND 8. Of the 48 pigs, 24 pigs (12 CHCF1 RS and 12 CHCF1 RR) were randomized for an experimental ETEC challenge trial blocked by CHCF1 genotype and body weight. We compared their fecal microbiota at early post-weaning (PND 24), prior to an ETEC F4ab challenge. Fig 1 shows an illustration of the study timeline.

### CHCF1 genotyping

Tail samples from tail docking at PND 4 were used to CHCF1 genotype the pigs according to Rydal and colleagues [16].

### Oxford Nanopore long-read sequencing of the bacterial 16S rRNA gene

#### Sample DNA extraction.

DNA extraction of samples was done using a slightly modified version of the standard protocol for FastDNA Spin kit for Soil (MP Biomedicals, USA) with the following exceptions: Swabs were washed thoroughly into the storage buffer, and then 500 μL of this solution along with 480 μL Sodium Phosphate Buffer and 120 μL MT Buffer added to a Lysing Matrix E tube. Bead beating was performed at 6 m/s for 4x40s [17]. Gel electrophoresis using Tapestation 2200 and Genomic DNA screentapes (Agilent, USA) was used to validate product size and purity of a subset of DNA extracts. DNA concentration was measured using Qubit dsDNA HS/BR Assay kit (Thermo Fisher Scientific, USA).

#### Sequencing library preparation.

25 ng of extracted DNA was used as template for PCR amplification of the bacteria 16S rRNA gene variable regions 1–8 (V1-V8). Each PCR reaction (50 μL) contained 0.5 mM dNTP mix, 0.01 units of Platinum SuperFi DNA Polymerase (Thermo Fisher Scientific, USA), and 500 nM of each forward and reverse primer in the supplied SuperFI Buffer. PCR was done with the following program: Initial denaturation at 98 °C for 3 min, 25 cycles of amplification (98 °C for 30 s, 62 °C for 20 s, 72 °C for 2 min) and a final elongation at 72 °C for 5 min. The forward and reverse primers used include custom 24 nucleotide barcode sequences attached to the sequences targeting the bacteria 16S rRNA gene V1-V8: [8F] AGRGTTYGATYMTGGCTCAG and [1391R] GACGGGCGGTGWGTRCA [18–21]. The resulting amplicon libraries were purified using the standard protocol for CleanNGS SPRI beads (CleanNA, NL) with a bead to sample ratio of 3:5. DNA was eluted in 25 μL of nuclease free water (Qiagen, Germany). Sequencing libraries were prepared from the purified amplicon libraries using the SQKLSK114 kit (Oxford Nanopore Technologies, UK) according to manufacturer protocol with the following modifications: 500 ng total DNA was used as input, and CleanNGS SPRI beads for library cleanup steps. DNA concentration was measured using Qubit dsDNA HS Assay kit (Thermo Fisher Scientific, USA). Gel electrophoresis using Tapestation 2200 and D1000/High sensitivity D1000 screentapes (Agilent, USA) was used to validate product size and purity of a subset of amplicon libraries.

#### DNA sequencing.

Circa 12 ng (~20 fmol) of the resulting sequencing library was loaded onto a MinION R10.4.1 flowcell and sequenced using the MinKNOW v22.03.6 software (Oxford Nanopore Technologies, UK). Sequencing reads were basecalled and demultiplexed with MinKNOW guppy v. 6.0.7 using the super accurate basecalling algorithm (config r10.4.1_450bps_sup.cfg) and custom barcodes.

#### Bioinformatic processing.

The SILVA 16S/18S rRNA 138 SSURef NR99 full-length database in RESCRIPt format was downloaded from the QIIME on 29 september 2022 [22–24]. DNA sequencing reads were filtered for length (320–2000 bp) and quality (phred score > 15) using the filtlong v0.2.1 tool. The filtered DNA reads were mapped to the SILVA 138.1 99% NR database entries using minimap2 v2.24 and further evaluated using samtools v1.14 [25,26]. The mapping results were filtered such that the observed alignment between the queried sequencing read and the database sequence mapped to deviated < 5% from the length of the sequencing read. Furthermore, results with a mapping quality score < 0.95, and low abundant observations making up < 0.01% of the total number mapped reads within each sample, were disregarded as a data denoising step. Taken together, this produced a so-called observed taxonomic unit (OTU) table. Samples yielding less than 8000 mapped DNA reads were disregarded in all subsequent analyses.

### Statistics

Data was analyzed and visualized using R version 4.3 [27]. P-values lower than 0.05 were considered significant and 0.1 were considered tendencies. Experimental groups were a group of CHCF1 RS pigs that were compared with a group of CHCF1 RR pigs at PND 8, PND 22, and PND 24.

Three samples had fewer than <8000 mapped reads and were excluded from the study leading to n = 48, 47 and 22 pig samples at PND 8, PND 22 and PND 24, respectively. Eight samples were available from the sows at PND 8. OTU count tables were rarefied to 8372 reads/sample before analysis of alpha and beta diversity.

The following R-packages were used for microbiota analysis and visualization: vegan [28], phyloseq [29], microbiome [30], ANCOMBC-2 [31], ggplot2 [32].

Differences in alpha diversity between CHCF1 genotypes were investigated using Shannon and Simpson diversity indices and number of observed OTUs (richness) at each timepoint and across timepoints. Data was analyzed using linear mixed models (lme4 [33], lmerTest [34]): at individual timepoints with CHCF1 genotype as fixed effect and litter as random effect, and longitudinal with CHCF1 genotype and age (scaled) as fixed effects and with pig and sow as random effects. Model residuals were checked for normal distribution using QQ-plots. The number of observed *E. coli* OTUs between genotypes were tested for differences at PND 8 using linear mixed models: with CHCF1 genotype as fixed effect and litter as random effect.

Differences in beta diversity between CHCF1 RS and CHCF1 RR groups were assessed using Canberra and binary jaccard distance matrices and analyzed with PERMANOVA at 999 permutations. Group differences between CHCF1 genotypes were assessed at each time point with litter included in the model as strata. Effect of litter was investigated at each timepoint in separate models with litter as the only explanatory variable. The betadisper function was used to check for multivariate homogeneity of group dispersions [35]. Data was visualized using non-metric multidimensional scaling (NMDS) plots.

Mean relative abundance was calculated at genus level and the most abundant genera were visualized.

Differential abundance analysis was performed with raw counts at OTU and genus level using ANCOM-BC2, where differences between CHCF1 genotypes were investigated at each timepoint accounting for litter as random effect. Prevalence cut was set to 10%, structural zeroes were detected, and p-values were adjusted using the Holm–Bonferroni method.

Differences in raw counts at species level for *Escherichia coli* and at family level of *Prevotellaceae, Lachnospiraceae, Ruminococcaceae* and *Lactobacillaceae* between CHCF1 genotypes at PND 8, were investigated using generalized linear mixed models (glmmTMB [36]), assuming negative binomial distribution, with litter as random effect. p-values were adjusted using the Holm–Bonferroni method.

## Results

### Alpha diversity

**Fig 2:**
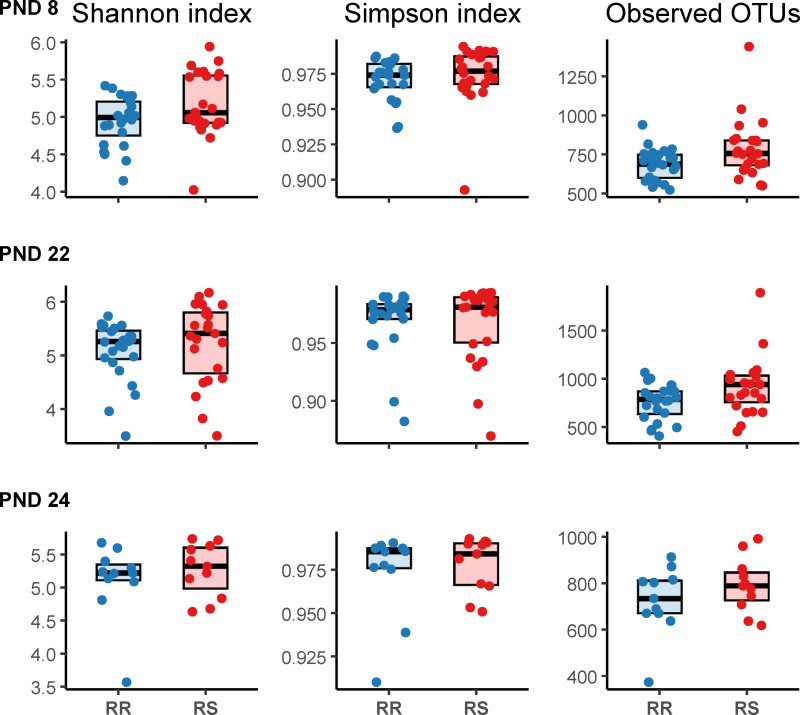
Alpha diversity boxplots by timepoint and CHCF1 genotype. The bold line represents the median. RR: homozygous resistant, RS: heterozygous susceptible. PND 8: early lactation (RR: n = 24 pigs, RS: n = 24 pigs). PND 22: late lactation/weaning (RR: n = 24 pigs, RS: n = 23 pigs). PND 24: two days after weaning at experimental facility (RR: n = 11 pigs, RS: n = 11 pigs). Data was first analyzed at each timepoint using linear mixed models with litter included as random effect. PND 8: Shannon index: coef: 0.21, 95% confidence interval (CI) [-0.01; 0.43], p = 0.05, Simpson index: coef: 0.003 95% CI [-0.006;0.01], p = 0.4, Observed number of OTUs: coef: 70.6, 95% CI [-11.0; 155.9], p = 0.08. PND 22: Shannon index: coef: 0.16, 95% CI [-0.23;0.53], p = 0.3, Simpson index: coef: -0.002, 95% CI [-0.02;0.01], p = 0.7, Observed number of OTUs: coef: 163.9, 95% CI [23.90;303.9], p = 0.02. PND 24: Shannon index: coef: 0.16, 95% CI [-0.22;0.54] p = 0.3, Simpson index: coef: 0.005, 95% CI [-0.01;0.02], p = 0.5, Observed number of OTUs: coef: 66.4, 95% CI [-44.6;177.5], p = 0.2.

Shannon and Simpson diversity and the number of observed OTUs were numerically higher in CHCF1 RS pigs compared to CHCF1 RR pigs at each timepoint (Fig 2).

*Analysis for difference between CHCF1 RS and RR genotypes across timepoints showed that the number of observed OTUs was significantly higher in pigs with CHCF1 RS genotype: 103, 95% CI [18.90; 192.76], p = 0.014. The observed number of OTUs increased significantly based on age: coef: 36.69, 95% CI [5.37; 67.79], p = 0.02. Shannon and Simpson indices were not significantly different between genotypes or based on age.*

**Fig 3:**
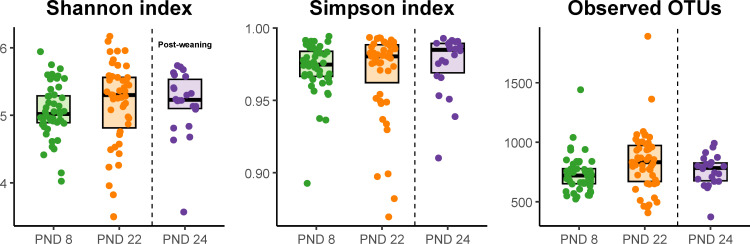
Boxplots showing alpha diversity development over time from early lactation to one day after weaning. Post-natal day (PND) 8 (n = 48 pigs), PND 22 (n = 47 pigs), PND 24 (n = 22 pigs). The bold line represents the median.

At PND 8, there were tendencies to differences in Shannon diversity (F-value: 3.8, p = 0.05) and number of observed OTUs (F-value: 3.1, p = 0.08), but differences in Simpson index were not significant (F-value: 0.57, p = 0.4). At PND 22, the number of observed OTUs was significantly higher in CHCF1 RS pigs (F-value: 5.48, p = 0.02), but neither Shannon nor Simpson indices were significantly different (F-value: 0.76, p = 0.3 and F-value: 0.09, p = 0.76, respectively). At PND 24, there were no significant differences between the two groups in neither Shannon and Simpson indices nor in the number of observed OTUs (F-values: 0.78, 0.46, 1.5 and p-values = 0.3, 0.5, 0.2, respectively). The number of observed OTUs was higher in the CHCF1 RS pigs compared to CHCF1 RR pigs across timepoints (F-value: 6.58, p = 0.01) and there was a also a significant increase with animal age (F-value = 5.4, p = 0.02). No significant differences between genotypes in Shannon (F-value: 2.38, p = 0.12) or Simpson indices (F-value = 0.04, p = 0.82) were observed across time. Alpha diversity increased with age in the preweaning period, and plateaued at PND 22 (Fig 3).

### Beta diversity

**Fig 4:**
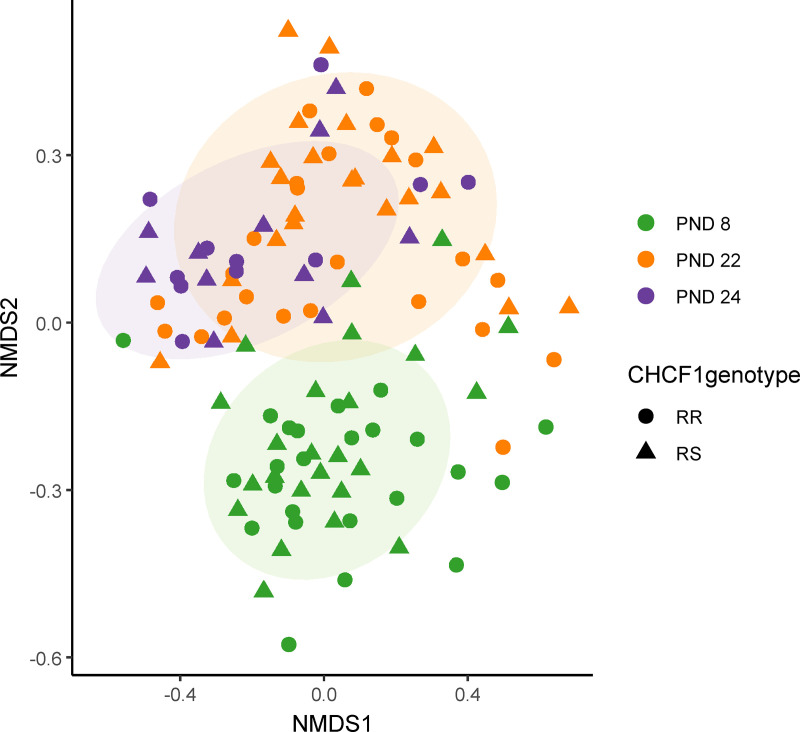
NMDS plot showing similarity of fecal bacterial communities based on age and CHCF1 genotype of pigs. RR: homozygous resistant, RS: heterozygous susceptible, PND: post-natal day. Generated from canberra distance matrix. Ellipses show 80% confidential areas assuming multivariate t-distribution. PND 8: early lactation, RR: n = 24 pigs, RS: n = 24 pigs. PND 22: late lactation, RR: n = 24 pigs, RS: n = 23 pigs. PND 24: one day after weaning at experimental facility, RR: n = 11 pigs, RS: n = 11 pigs. There were no significant differences between CHCF1 RR and RS pigs at any timepoints.

**Fig 5:**
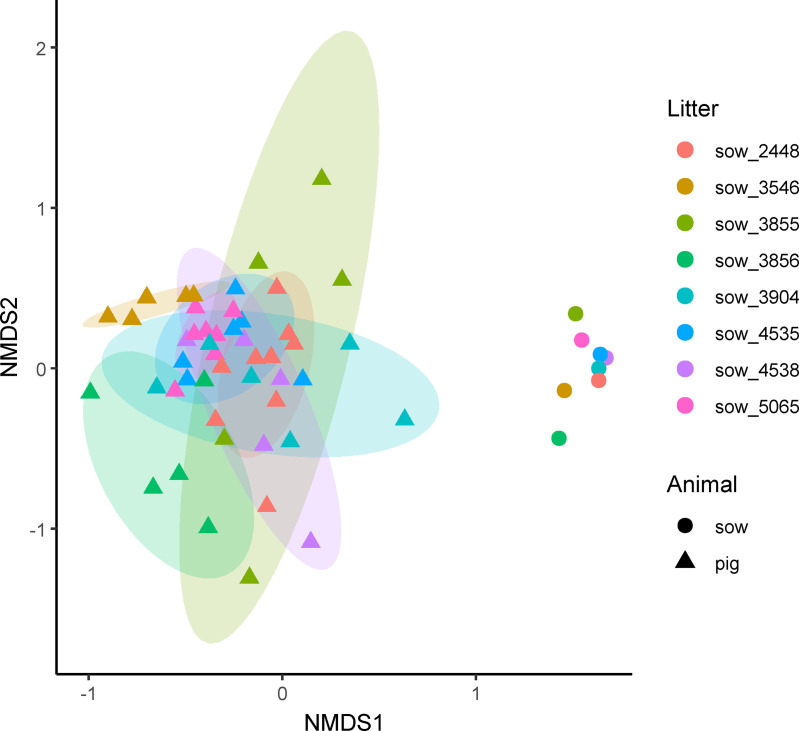
NMDS plot showing similarity of fecal bacterial communities based on litter of origin. NMDS plot was generated from canberra distance matrix and shows the overall fecal bacterial communities of sows (n = 8 sows) and their litters at PND 8 (n = 48 pigs). Ellipses show 80% confidential areas assuming multivariate t-distribution. Sow 3546 was treated with sulfa/TMP against MMA and all pigs of the litter with Apramycin against diarrhea at PND 3-5. There were significant differences in overall bacterial communities between pigs of different litters at PND 8 (R^2^ = 0.2, p = 0.001).

Overall bacterial communities did not differ significantly between CHCF1 RS and CHCF1 RR pigs at any of the three timepoints (Fig 4) and were more similar within than between litters (Fig 5).

Differences in beta diversity between litters persisted throughout the pre-weaning period (PND 22: R^2^ = 0.23, p = 0.001), and continued to be pronounced after weaning, even as litters had been mixed at the experimental facility (PND 24: R^2^ = 0.4, p = 0.001). There were no significant differences in dispersion between genotypes (Figs 4 and S2) or litters (Figs 5 and S3).

### Differential abundance

**Fig 6:**
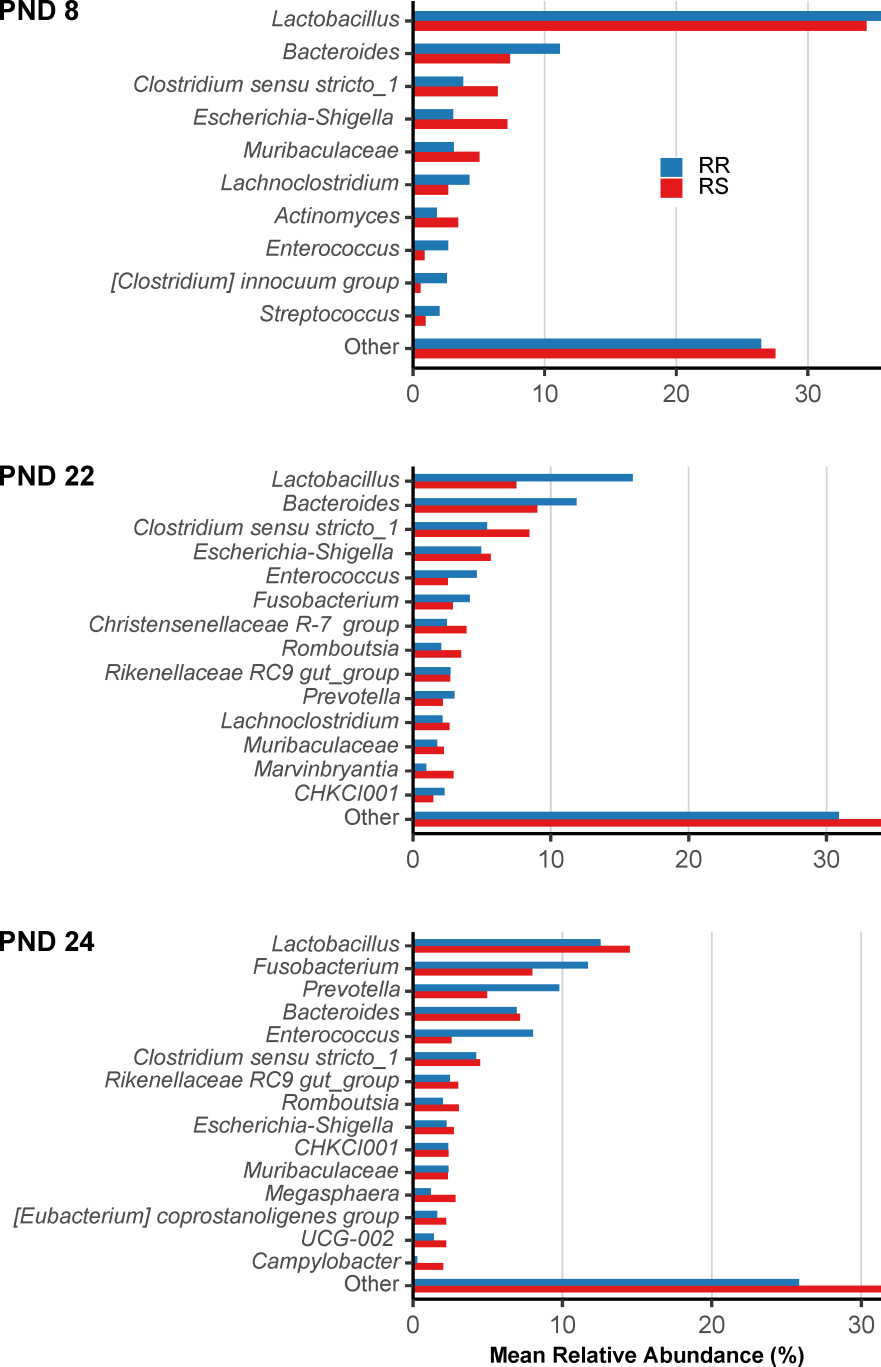
An overview of the most abundant bacterial genera of CHCF1 RR (homozygous resistant) and RS (heterozygous susceptible) pigs. Tested at early lactation (post-natal day (PND) 8, RR: n = 24 pigs, RS: n = 24 pigs), late lactation (PND 22, RR: n = 24 pigs, RS: n = 23 pigs), and one day after weaning (PND 24, RR: n = 11 pigs, RS: n = 11 pigs). Taxa with <2% relative abundance pooled into “other”. PND 8: accumulated unassigned taxa at genus level represent 3.48% (RR) and 3.43% (RS). PND 22: accumulated unassigned taxa at genus level represent 4.79% (RR) and 8.55% (RS). PND 24: accumulated unassigned taxa at genus level represent 4.94% (RR) and 4.14% (RS).

Pre-weaning, *Lactobacillus spp.*, *Bacteriodes spp., Escherichia/Shigella spp.* and *Clostridium sensu stricto spp.* were the most abundant genera across genotypes (Fig 6).

After weaning, mean relative abundance of *Prevotella spp.* increased making it one of the most abundant genera and decreases in *Escherichia/Shigella spp.* were found for both genotypes (Fig 6). *Escherichia coli* was of special interest as pigs with CHCF1 susceptible genotype may have phenotypic expression of F4ab/ac receptors. At species level, *Escherichia coli* had numerically higher mean relative abundance in CHCF1 RS pigs compared to CHCF1 RR pigs, particularly at PND 8 (6.1% vs 2.4%, respectively) (S4 Fig), but not significantly higher when assessing abundance in raw counts with generalized linear mixed models. We also found numerically higher numbers of different OTUs identified as *E. coli* in CHCF1 RS pigs compared to CHCF1 RR pigs at PND 8 (CHCF1 RS: mean = 54.2, median = 7, Q1 = 2.5, Q3 = 52.2, max = 362, min = 0, and CHCF1 RR: mean = 28.5, median = 5.5, Q1 = 0.75, Q3 = 29.5, max = 192, min = 0). However, it was not statistically significant. The total number of possible OTUs identified as *E. coli* in the study were 780.

No significant differentially abundant bacteria were detected between genotypes using ANCOMBC-2 at neither genus or OTU level at any time point. We did not find significant differences in abundance of families *Prevotellaceae, Lachnospiraceae, Ruminococcaceae* and *Lactobacillaceae* between genotypes at PND 8, when we assessed these taxa individually using generalized linear mixed models.

## Discussion

We investigated if there were differences in certain taxa or diversity measures of the fecal microbiota that could contribute to explain why CHCF1 susceptible pigs developed diarrhea after inoculation with ETEC F4ab, while CHCF1 resistant pigs did not [16]. The only statistically significant differences were slightly higher fecal microbiota richness (number of observed OTUs) for CHCF1 susceptible pigs, a feature that was consistent from early lactation till just after weaning. MUC4 susceptible genotypes have previously been associated with lower bacterial diversity in jejunum content using 16S rRNA gene-targeted denaturing gradient gel electrophoresis fingerprinting analysis [13], but this was not confirmed in a later study [11]. Our microbiota analysis was performed on fecal material and conducted with full length sequencing technology of the 16S rRNA gene, providing higher resolution. We speculate that our finding of higher alpha diversity could be explained by F4ab/ac receptor expression in the CHCF1 susceptible pigs [14] enabling more bacterial strains, such as F4ab/ac fimbriae-carrying *E. coli*, to adhere and proliferate in the gut. While we observed numerically higher abundance as well as number of observed *E. coli* OTUs in CHCF1 RS pigs, it is important to emphasize that these differences were not statistically significant, which allows only for speculation on this finding. Full length sequencing of the 16S rRNA gene can potentially achieve resolution at species and even strain level as demonstrated for *E. coli* [37]. A higher number of different *E. coli* strains (matching to different sequences in the SILVA database) in CHCF1 RS pigs could be part of the reason why we saw higher numbers of observed OTUs in these pigs. However, as the differences found in our study failed to reach the significance threshold, this remains a compelling hypothesis for further study. Furthermore, the specific trait behind CHCF1 genotype that explains susceptibility to ETEC F4ab/ac remains unknown [14]. In theory, it could be associated with adhesion and survival of other species of bacteria, which could explain the higher alpha diversity in CHCF1 RS pigs. As examples of other traits that may be affected by the F4ab/ac receptor locus, MUC4 genotypes were found to differ in gene expression related to intestinal immune function and mucosal integrity [11,38]. Otherwise, we found that pigs from the two CHCF1 genotypes had quite similar fecal microbiotas pre- and shortly post-weaning. Other recent research in pig herds suggests that PWD cases can have quite similar fecal microbiotas pre-weaning compared to healthy controls [39,40]. We also included a sample for microbiota comparison just after weaning to learn how weaning affected the stability of the microbiota between ETEC F4ab susceptible and resistant pigs. However, we did not find any significant differences around 24 hours after weaning between the groups. The finding that the early-life fecal microbiota may not be useful for assessing susceptibility to PWD contradicts the studies of Dou and colleagues [4] and Karasova and colleagues [5]. We already discussed possible reasons for this in our previous study [39].

The most abundant genera found before and after weaning in this study were highly similar to what we found previously [39], even though we used a different lab, sequencing technology and conducted the study a year later with pigs from the same herd. *Escherichia spp., Lactobacillus spp*., and *Bacteriodes spp.* were the most abundant genera at the pre-weaning timepoints, whereas a decrease in mean relative abundance of *Escherichia spp.* and an increase in *Prevotella spp.* was observed after weaning. This dynamic is also in accordance with what has been described earlier [41–43].

The most significant finding of our study was the large effect of litter in our statistical analysis. It makes sense that the microbes in the pen environment and from the mother sow [44,45] have a large influence on shaping the piglet gut microbiota. However, these factors are often not accounted for in the statistical analysis of microbiota data in the scientific literature within our field. Interestingly, the large litter effects persisted after weaning, transport and mixing pigs in new environments. Our findings imply that litter effects should be taken into account in experimental designs and statistical analysis when studying piglets and newly weaned pigs. In future studies, it would be interesting to investigate the duration of these litter effects in the period from weaning and onwards, and to investigate if different batches of pigs born from the same mother also share similarities. Furthermore, if the pen environment and mother play a great role in shaping the gut microbiota, this also implies that the pen environment and perhaps the maternal microbiota could potentially affect gut health outcomes for the litter [46].

In conclusion, in pigs from a single herd, differences between CHCF1 genotypes were minor and only with statistical significance in terms of higher alpha diversity in CHCF1 RS pigs. Littermates had more similar fecal microbiotas to each other compared with pigs from different litters from early-life (PND 8) and till one day after weaning (PND 24). We did not find the pre-weaning or early post-weaning fecal microbiota to contain taxa or diversity markers that could explain why CHCF1 RS pigs developed ETEC F4ab-associated diarrhea in contrast to CHCF1 RR pigs.

## Supporting information

S1 Fig Randomization scheme. The randomization process was performed in three steps. We had genotyped all female pigs with birthweight ranging from 1–2 kg from a total of 16 sows, which included all the multiparous sows of the farrowing batch at the herd. Step 1, 24 CHCF1 RS pigs were randomized from the population of all available CHCF1 RS female pigs from the 16 litters. This randomization led to 24 CHCF1 RS pigs distributed between 8 litters. Step 2, 24 CHCF1 RR pigs were randomized out of the total population of female CHCF1 RR pigs originating from the same 8 litters, where the 24 CHCF1 RS pigs had been located. Step 3, we listed the 24 CHCF1 RS and 24 CHCF1 RR pigs from lowest to highest weaning weight and randomized blocked by weight and genotype, a subset of 12 CHCF1 RS and 12 CHCF1 RR pigs for an infection trial. Created with BioRender.com.
(TIF)

S2 Fig Binary Jaccard distance matrix generated NMDS plot showing microbiota similarities between CHCF1 genotypes at PND 8, 22 and 24. (TIF)

S3 Fig NMDS plot, based on binary Jaccard distance matrix, showing similarity of fecal bacterial communities based on litter of origin. The plot illustrates within litter similarities of fecal microbiotas of pigs at PND 8 (n = 48 pigs) and fecal microbiotas of their mothers (n = 8).
(TIF)

S4 Fig An overview of the most abundant bacterial taxa of CHCF1 RR (homozygous resistant) and RS (heterozygous susceptible) pigs. Taxa identified at species level or closest identifiable level. Taxa with <2% relative abundance pooled into “other”.
(TIF)
