# Correction: The early-life fecal microbiota is associated with litter of origin but not with susceptibility to ETEC F4ab-mediated post-weaning diarrhea in CHCF1 genotyped pigs

**DOI:** 10.1371/journal.pone.0346785

**Published:** 2026-04-07

**Authors:** The PLOS One Staff

In the Results, section, the following information from the caption for Fig 2 was incorrectly included:

Analysis for difference between CHCF1 RS and RR genotypes across timepoints showed that the number of observed OTUs was significantly higher in pigs with CHCF1 RS genotype: 103, 95% CI [18.90; 192.76], p = 0.014. The observed number of OTUs increased significantly based on age: coef: 36.69, 95% CI [5.37; 67.79], p = 0.02. Shannon and Simpson indices were not significantly different between genotypes or based on age.

Please see the complete, correct Fig 2 caption here.

The publisher apologizes for the errors.

**Funding**

**Fig 2:**
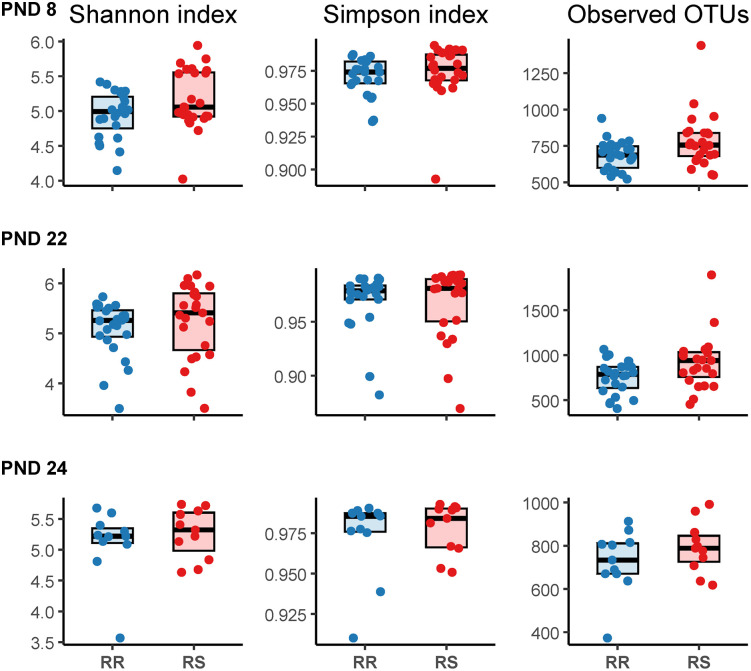
Alpha diversity boxplots by timepoint and CHCF1 genotype. The bold line represents the median. RR: homozygous resistant, RS: heterozygous susceptible. PND 8: early lactation (RR: n = 24 pigs, RS: n = 24 pigs). PND 22: late lactation/weaning (RR: n = 24 pigs, RS: n = 23 pigs). PND 24: two days after weaning at experimental facility (RR: n = 11 pigs, RS: n = 11 pigs). Data was first analyzed at each timepoint using linear mixed models with litter included as random effect. PND 8: Shannon index: coef: 0.21, 95% confidence interval (CI) [−0.01; 0.43], p = 0.05, Simpson index: coef: 0.003 95% CI [−0.006;0.01], p = 0.4, Observed number of OTUs: coef: 70.6, 95% CI [−11.0; 155.9], p = 0.08. PND 22: Shannon index: coef: 0.16, 95% CI [−0.23;0.53], p = 0.3, Simpson index: coef: −0.002, 95% CI [−0.02;0.01], p = 0.7, Observed number of OTUs: coef: 163.9, 95% CI [23.90;303.9], p = 0.02. PND 24: Shannon index: coef: 0.16, 95% CI [−0.22;0.54] p = 0.3, Simpson index: coef: 0.005, 95% CI [−0.01;0.02], p = 0.5, Observed number of OTUs: coef: 66.4, 95% CI [−44.6;177.5], p = 0.2. Analysis for difference between CHCF1 RS and RR genotypes across timepoints showed that the number of observed OTUs was significantly higher in pigs with CHCF1 RS genotype: 103, 95% CI [18.90; 192.76], p = 0.014. The observed number of OTUs increased significantly based on age: coef: 36.69, 95% CI [5.37; 67.79], p = 0.02. Shannon and Simpson indices were not significantly different between genotypes or based on age.
