# Supplementary figures and images for: The early-life fecal microbiota is associated with litter of origin but not with susceptibility to ETEC F4ab-mediated post-weaning diarrhea in CHCF1 genotyped pigs
Source: PLoS One. 2025 May 29;20(5):e0323875. doi: 10.1371/journal.pone.0323875 (PMC12121822; doi:10.1371/journal.pone.0323875)

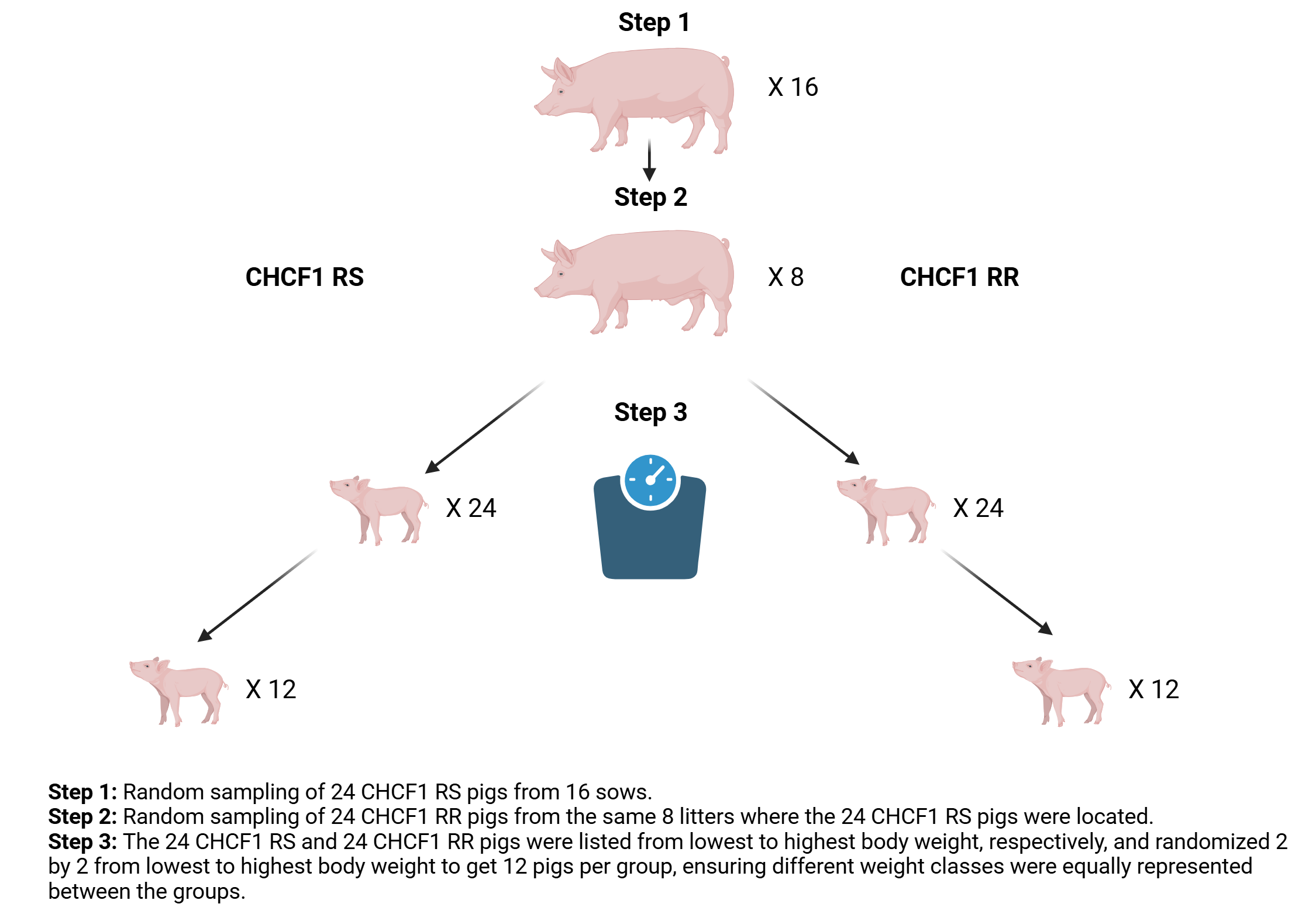

Supplement: S1 Fig — The randomization process was performed in three steps. We had genotyped all female pigs with birthweight ranging from 1–2 kg from a total of 16 sows, which included all the multiparous sows of the farrowing batch at the herd. Step 1, 24 CHCF1 RS pigs were randomized from the population of all available CHCF1 RS female pigs from the 16 litters. This randomization led to 24 CHCF1 RS pigs distributed between 8 litters. Step 2, 24 CHCF1 RR pigs were randomized out of the total population of female CHCF1 RR pigs originating from the same 8 litters, where the 24 CHCF1 RS pigs had been located. Step 3, we listed the 24 CHCF1 RS and 24 CHCF1 RR pigs from lowest to highest weaning weight and randomized blocked by weight and genotype, a subset of 12 CHCF1 RS and 12 CHCF1 RR pigs for an infection trial. Created with BioRender.com. (TIF) [file pone.0323875.s001.tif]

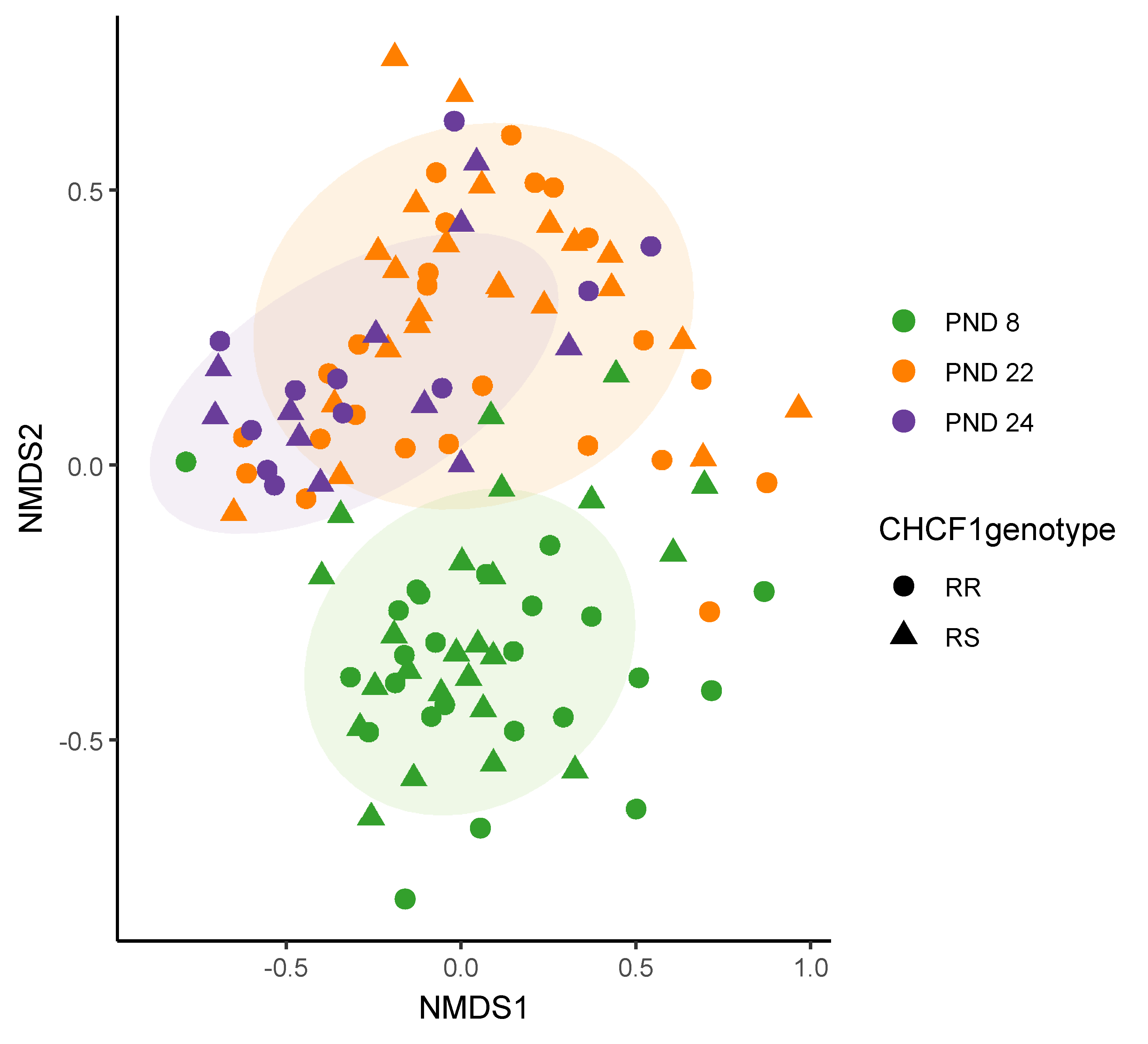

Supplement: S2 Fig — (TIF) [file pone.0323875.s002.tif]

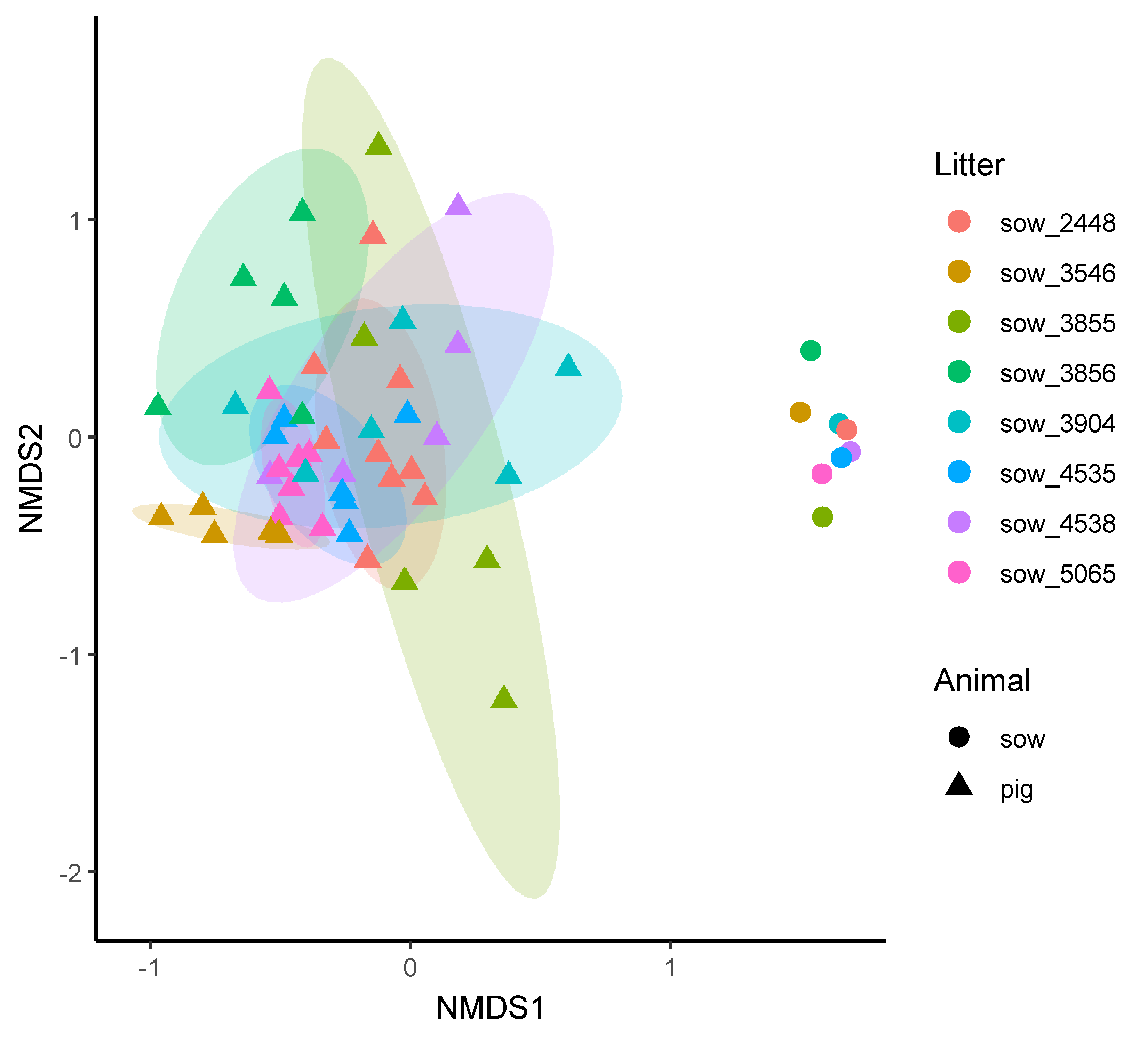

Supplement: S3 Fig — The plot illustrates within litter similarities of fecal microbiotas of pigs at PND 8 (n = 48 pigs) and fecal microbiotas of their mothers (n = 8). (TIF) [file pone.0323875.s003.tif]

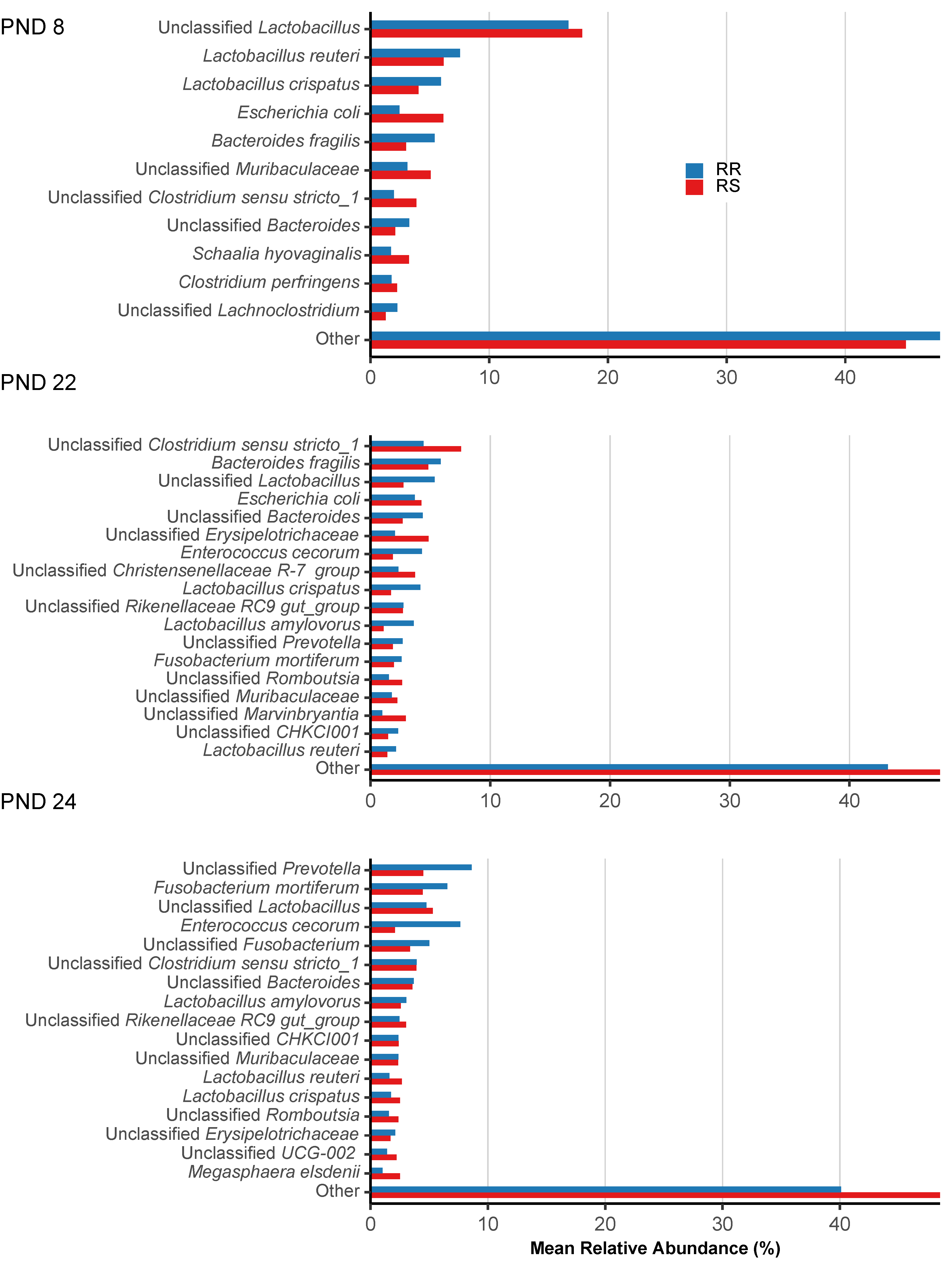

Supplement: S4 Fig — Taxa identified at species level or closest identifiable level. Taxa with <2% relative abundance pooled into “other”. (TIF) [file pone.0323875.s004.tif]
