# Supplementary material for: The early-life fecal microbiota is associated with litter of origin but not with susceptibility to ETEC F4ab-mediated post-weaning diarrhea in CHCF1 genotyped pigs
Source: PLoS One. 2025 May 29;20(5):e0323875. doi: 10.1371/journal.pone.0323875 (PMC12121822; doi:10.1371/journal.pone.0323875)
Supplement: Attachment — Submitted filename: review comments.pdf [file pone.0323875.s005.pdf]

This paper investigates an important and topical topic. The tests are well-planned, and the data is strong. However, there are a few areas where extra clarification and refinement may help the conclusions stand out. Clearer explanations for non-significant results, as well as a more accessible exposition of some technical procedures, would help to improve the manuscript. Overall, this is a valuable addition. However, I would advise some revision before it is published.

Key strength: The manuscript is well-organized, with a logical flow and clear sections. The work addresses an important issue in pig husbandry, with implications for both veterinary science and agricultural practice.

Areas for Improvement:

Line 22-24: The genetic vulnerability to ETEC F4ab/ac statement in the introduction would benefit from further context regarding the significance of these discoveries in larger pig production systems or possible human uses. For clarity, think about going into more detail.

Line 25-30: The study population and timing are clearly described, yet there may have been a more seamless transition from discussing genetic susceptibility to outlining the experimental challenge. You can briefly discuss the significance of PND 8, PND 22, and PND 24 time points for the study.

Line 117-177: There is a lot of technical jargon in this section. Think about elucidating terminology like “filtlong” and “minimap2” in further detail or simplifying them for readers who might not be familiar with bioinformatic tools. Accessibility and readability would both be enhanced by this.

Line 220-231: The explanation of the alpha diversity analysis results is clear, yet it would be beneficial to draw attention to the statistical insignificance of several measurements, such as the p-values of 0.05 and 0.4 for the Shannon and Simpson indices, respectively. This keeps results that are close to significance but fall short of the cutoff from being overinterpreted.

Line 310-317: When discussing the higher alpha diversity in CHCF1 susceptible pigs, the potential explanations are well-presented, but further clarification about the statistical insignificance of E. coli abundance (line 316-317) should be highlighted. Emphasize that these trends were not statistically significant to avoid confusion.

Line 221-239: You mention trends in Simpson indices and Shannon diversity in the discussion of alpha diversity. When using the term "tendencies" in scientific reporting, be sure to define it precisely. While making a suggestion of a potential trend, you might clearly say that these numbers did not satisfy the significance threshold.

Line 325-327: Although the explanation of the possible roles played by various strains of E. coli is informative, it might be more precise. Even if there were higher OTU numbers, the differences were not statistically significant, thus you could still have a compelling hypothesis for further study.
