# Supplementary material for: The early-life fecal microbiota is associated with litter of origin but not with susceptibility to ETEC F4ab-mediated post-weaning diarrhea in CHCF1 genotyped pigs
Source: PLoS One. 2025 May 29;20(5):e0323875. doi: 10.1371/journal.pone.0323875 (PMC12121822; doi:10.1371/journal.pone.0323875)
Supplement: Attachment — Submitted filename: Response to Reviewers.docx [file pone.0323875.s007.docx]

Reviewer 1:

Dear reviewer 1,

Thank you for your valuable time and well-considered input. Please find our elaborated response below in red.

Lines 91-111: The Experimental design is poorly described. This sounds like a 2x2 factorial, so it should be declared that way.

The study was a longitudinal, observational study with stratified randomization. We did this randomization technique to ensure that the CHCF1 RS and CHCF1 RR groups were balanced in terms of numbers of pigs with susceptible and resistant genotypes. The subset of pigs that were followed after weaning prior to an ETEC F4 challenged were randomized stratified by both CHCF1 genotype and body weight to ensure that the groups were also balanced in terms of body weight at the experimental facility (**Fig 1, SI 1 Fig**).

More details can now be found in the manuscript, **lines 104-122**.

Line 91: replace "randomized" with "selected"

Updated **line 106**: “randomized in blocks”

We believe “randomized” is the more correct term, as the study population was indeed chosen by randomizing available pigs and not merely “selected”, which we believe can be misread as convenience sampling.

Line 93: How many pigs were selected from each sow? Were the sows profiled?

Ranging from 5-9 pigs (**Fig 5**). Information added to “Animals” section in the methods, **lines 87-88.**

The sows were not genotyped.

Lines 93-94: How were pigs identified as RS or RR?

Pigs were identified as RS or RR based on the CHCF1 allele marker, where:

CHCF1 (resistant allele = C)

ACATAGAGACTTTCTAGCAAAGTTGCTAGGAGAGCAAATCTTAAAAGTTCT[C/A]AAGG

CGGAGTTCCCATTGTGGCACAGTGGAAATGGATCTGACTGGGAAC

More information on the genotyping can be found in:

https://doi.org/10.1016/j.vetmic.2023.109771.

Lines 94-95: This seems out of order.

We have updated the sentence to clarify what was compared. Updated **line 109-110**:

“The fecal microbiotas of the 24 CHCF1 RS and 24 CHCF1 RR pigs were compared at early lactation (PND 8) and at late lactation (PND 22).”

Line 97: Where and how were pigs housed?

At the experimental facility, the 12 CHCF1 RS and 12 CHCF1 were housed in two separate rooms with one pen per room. We did this and used strict hygiene standards to avoid cross contamination of the challenge strain between groups. We have updated “Animals” section of the methods for clarity, **lines 95-96**.

Details on the housing conditions at the experimental facility has been described in detail in the paper describing the infection trial^[1]^.

Lines 100-101: This does not belong here, rather Discussion.

We have removed this information from the experimental design section.

Lines 101-102: This only accounts for 24 of 48 pigs.

We were referring only to the subpopulation of pigs that were part of the subsequent ETEC challenge trial. The information has been removed from the experimental design section, as it already appears in the discussion, **lines 319-321.**

Line 106: What were the treatment groups exactly?

We followed a group of CHCF1 RS pigs and a group of CHCF1 RR pigs from the same herd and sows that had the same conditions in terms of feeding, housing and management. They only differed in CHCF1 genotype.

A visualization of the microbiota study design is available in **Fig 1**. Briefly, we first looked at differences in fecal microbiota composition between 24 CHCF1 RS and 24 RR pigs at two pre-weaning timepoints at the herd (PND 8 and PND 22). Then a subset of 12 CHCF1 RS and 12 CHCF1 RR pigs were randomized for an experimental challenge trial, where we looked at fecal microbiota difference 1 day after weaning, just before an experimental challenge with ETEC F4ab. In the current microbiota study, we only followed the 12 CHCF1 RS and 12 CHCF1 RR pigs till PND 24 (1 day after weaning) at the experimental facility.

The subsequent infection trial included the two groups described above: CHCF1 RS (n=12) and CHCF1 RR (n=12). The two groups received the same feed and ETEC F4ab challenge in terms of strain, dose and timing. They differed only in genotype. The response to ETEC inoculation in terms of diarrhea development and clinical condition has been described in a paper describing the infection trial^1^. In the paper on the infection trial^1^, you can find that all the 12 CHCF1 RS developed ETEC F4ab –mediated diarrhea whereas none of the 12 CHCF1 RR pigs did. This is mentioned in the discussion (**lines 319-321**) as it shows that pigs in our microbiota study were not just genetically susceptible/resistant, but were also confirmed to be clinically susceptible/resistant to ETEC F4ab.

Line 108: Were all the pigs challenged with ETEC?

Only the subset of 12 CHCF1 RS and 12 CHCF1 RR pigs that were included in the experimental infection trial (**Fig 1**), **lines 111-115.**

Line 119: Provide at least some details of the methods.

The method on Oxford nanopore long-read sequencing of the bacterial 16S rRNA gene have been updated for improved accessibility for readers. **Lines 127-179**

Line 122: insert "Quality control" before abbreviation

Same as above.

Lines 123-124: Was it <10000 or <8000?

Below 8000.

Line 128: Was the protocol from a published study or the manufacturer?

Manufacturer.

https://www.mpbio.com/us/116560000-fastdna-spin-kit-for-soil-samp-cf?srsltid=AfmBOoqc8wzg4tP4fxydJIsJbPlR04qCFuVlO4e90CeWMIdlNiGP95g7

Line 138: Is the description of the custom protocol the text that follows?

Yes.

Line 182: What were the experimental groups? Were all the factors and interactions considered?

To clarify experimental groups are added to the statistical method, **lines 182-184.**

With great effort, the study was designed to minimize potential confounding factors (**lines 85-96**) thereby making it possible to simplify analysis.

For example,

1) By rearing pigs with their mother sow in the pen that they were born in at the farrowing unit. Thereby, we avoided cross-fostering which was a potential confounder for the fecal microbiota.

2) At birth, only pigs weighing between 1-2 kg were included. Thereby we avoided “outlier pigs” with low or very high birthweight. Now described in **line 88**.

3) We only included female pigs to avoid potential sex differences.

4) Pigs had the same housing, management and feeding conditions.

5) Pigs that were randomized for the infection trial were stratified by weaning weight to ensure that the same weight classes were equally represented between the CHCF1 RS and CHCF1 RR group.

6) We did not include litters from gilts.

Given the design of the study, it was appropriate to account for the random effect of litter (littermates cannot be seen as completely independent in terms of microbiota composition) in addition to the explanatory variable of interest (CHCF1 genotype) in analysis of alpha and beta diversity as well as in differential abundance analysis.

Line 186: insert "Operational taxonomic unit" before abbreviation

Now appears for the first time in in **lines 177-178.**

Line 199: Define the groups being considered.

“Differences in beta diversity between groups were assessed using Canberra and binary jaccard distance matrices and analyzed with PERMANOVA at 999 permutations.”

Has been updated to

“Differences in beta diversity between CHCF1 RS and CHCF1 RR groups were assessed using Canberra and binary jaccard distance matrices and analyzed with PERMANOVA at 999 permutations.”

**Lines 200-202**

Line 222: What were the responses of the pigs not challenged with ETEC?

All the 12 CHCF1 RS and 12 CHCF1 RR pigs that were included in the infection trial were challenged with the same dose of an ETEC F4ab strain. Since all were challenged, we cannot say anything about responses of pigs not challenged with ETEC.

The additional 12 CHCF1 RS and 12 CHCF1 RR that remained at the supplying herd were not continuously followed in the nursery.

Line 236: What was the comparison?

CHCF1 RS pigs were compared with CHCF1 RR pigs across time.

Updated for clarification, **line 237:**

“Analysis for difference between CHCF1 RS and RR genotypes across timepoints…”

Line 242: What was the comparison?

All pig samples, irrespective of genotype, are shown in boxplots at the three different timepoints (PND 8, PND 22 and PND 24) to visualize the development in alpha diversity across time (**Fig 3**).

Lines 307-end: The Discussion must be rewritten considering the updated statistical analysis.

Thank you for the comment. Given our explanation to your previous question “Line 182: What were the experimental groups? Were all the factors and interactions considered?”, we do not see a need for reanalyzing the data.

Reviewer 2:

Dear reviewer 2,

Thank you for your important insights and valuable time. Please find our elaborated response below in red.

The manuscript delivers a good study with well-executed experiments and unambiguous conclusions. However, it would benefit from putting a stronger emphasis on non-significant findings in the results section…

We agree with your point and have updated the results section to further increase transparency to the reader. **Lines 242-254**.

…and simplifying some of the technical terminology in the methods to improve accessibility.

We have simplified technical terminology in the part of the methods describing Oxford nanopore long-read sequencing of the bacterial 16S rRNA gene to improve reader accessibility. **Lines 127-179**

Furthermore, focusing on the implications of the litter effect in the discussion would increase the significance of the findings.

We have added thoughts on implications of the litter effect to the discussion, **lines 369-377**.

Editor comments:

Dear Dr. Mohamed O Ahmed,

Thank you for your important comments and time spend to help improve our manuscript. Please find our elaborated response below in red.

**Areas for Improvement:**

Line 22-24: The genetic vulnerability to ETEC F4ab/ac statement in the introduction would

benefit from further context regarding the significance of these discoveries in larger pig

production systems or possible human uses. For clarity, think about going into more detail.

We have elaborated in the introduction of the abstract for further clarity. **Lines 20-26.**

Line 25-30: The study population and timing are clearly described, yet there may have been

a more seamless transition from discussing genetic susceptibility to outlining the

experimental challenge. You can briefly discuss the significance of PND 8, PND 22, and

PND 24 time points for the study.

We have elaborated the method part of the abstract to address your point. **Lines 27-35.**

Line 117-177: There is a lot of technical jargon in this section. Think about elucidating

terminology like “filtlong” and “minimap2” in further detail or simplifying them for readers

who might not be familiar with bioinformatic tools. Accessibility and readability would both

be enhanced by this.

We have simplified technical terminology in the part of the methods describing Oxford nanopore long-read sequencing of the bacterial 16S rRNA gene to improve readability. **Lines 127-179**

Line 220-231: The explanation of the alpha diversity analysis results is clear, yet it would be

beneficial to draw attention to the statistical insignificance of several measurements, such

as the p-values of 0.05 and 0.4 for the Shannon and Simpson indices, respectively. This

keeps results that are close to significance but fall short of the cutoV from being

overinterpreted.

We have elaborated on the non-significant results in alpha diversity to further increase transparency to the reader. **Lines 242-254**.

Line 310-317: When discussing the higher alpha diversity in CHCF1 susceptible pigs, the

potential explanations are well-presented, but further clarification about the statistical

insignificance of E. coli abundance (line 316-317) should be highlighted. Emphasize that

these trends were not statistically significant to avoid confusion.

We have updated the discussion to emphasize that the numbers and abundances of *E. coli* were not statistically different. **Lines 331-334.**

Line 221-239: You mention trends in Simpson indices and Shannon diversity in the

discussion of alpha diversity. When using the term "tendencies" in scientific reporting, be

sure to define it precisely. While making a suggestion of a potential trend, you might clearly

say that these numbers did not satisfy the significance threshold.

Thank you for the comment. We used the term “tendencies” if p-values were below 0.1 but failed to satisfy the significant threshold set to below 0.05. We defined p-values below 0.1 as tendencies in the statistical method section, **line 182**.

Line 325-327: Although the explanation of the possible roles played by various strains of E.

coli is informative, it might be more precise. Even if there were higher OTU numbers, the

diVerences were not statistically significant, thus you could still have a compelling

hypothesis for further study.

We agree with your comment and hope that our updated discussion helps to clarify this point, **lines 339-340.**

1. Complete association between CHCF1 genotype and enterotoxigenic Escherichia coli F4ab-associated post-weaning diarrhea in a pig challenge trial. https://doi.org/10.1016/j.vetmic.2023.109771.
