# Supplementary material for: The early-life fecal microbiota is associated with litter of origin but not with susceptibility to ETEC F4ab-mediated post-weaning diarrhea in CHCF1 genotyped pigs
Source: PLoS One. 2025 May 29;20(5):e0323875. doi: 10.1371/journal.pone.0323875 (PMC12121822; doi:10.1371/journal.pone.0323875)
Supplement: Attachment — Submitted filename: Response_to_Reviewers_auresp_2.docx [file pone.0323875.s008.docx]

Response to Reviewers

Dear reviewer 3,

Thank you for your well-considered comments and time spend to help improve our manuscript. Please find our elaborate response below in red.

- I believe that this 2022 article in PlosONE on "Gut microbiome modifications over time when removing in-feed antibiotics from the prophylaxis of post-weaning diarrhea in piglets" can be beneficial for the Introduction or Discussion of your work (Cremonesi et al. 2022, PLoS ONE 17(3): e0262199. <https://doi.org/10.1371/journal.pone.0262199>)

Thank you for the reference, added line 362.

- it would be much better the place figures and tables within the text, otherwise the revision process becomes more complicated, and the reading experience is inconvenienced

Thank you for the comment and sorry for the inconvenience, however we must follow journal submission guidelines that state under “Figures”:

“Do not include figures in the main manuscript file. Each figure must be prepared and submitted as an individual file.”

- https://journals.plos.org/plosone/s/submission-guidelines

- figures are at low resolution hence not easy to read/assess

We have reuploaded figures with increased resolution to enhance reading experience.

Introduction

------------

- I suggest that you spell out the abbreviations -e.g. ETEC, PWD- the first time you introduce them in the main manuscript; not all readers will necessarily read the abstract as the first thing

Corrected, line 51.

L99: what are humane endpoints? Please be a little more specific

Thank you for the comment.

We described humane endpoints in detail in our paper on the infection trial:

“The pigs were scored clinically twice a day and assessment included wet perianal fecal staining, body condition score, signs of dehydration (rough hair coat, sunken eyes and flanks, skin turgor by pinch skinfold test), cyanosis and affected mental status (lay down a lot, had to be encouraged to move). Temperature and body weight was measured once daily and on indication. In addition, the pigs were clinically assessed every 3 h and on indication after inoculation until the end of the study period according to Additional file 1. In case of a clinical score 3, pigs would be monitored regularly and if they did not improve within 3 h they would be euthanized for humane reasons.”

- Quote. Rydal MP, Jørgensen CB, Gambino M, Poulsen LL, Nielsen JP. Complete association between CHCF1 genotype and enterotoxigenic Escherichia coli F4ab-associated post-weaning diarrhea in a pig challenge trial. Vet Microbiol. 2023 Jul;282:109771. doi: 10.1016/j.vetmic.2023.109771. Epub 2023 May 3. PMID: 37150059.

We reference to this article in line 103 so readers can find all information on humane endpoints that has already been described and published.

Methods

-------

L176-177: "an abundance count of the database entries mapped to, combined with their database-derived taxonomic assignment" : this sentence is quite convoluted and not easy to understand, please rephrase

Rephrased to:

“Taken together, this produced a so-called observed taxonomic unit (OTU) table.”

Line 176.

L181-182: you did not use any strategy to check for potentially spurious results? (p-values may come up as significant merely by chance)

We used the Holm-Bonferonni method to adjust p-values. Line 212-213 and 218.

L185: mapped reads

Corrected, line 184.

L186: why this dramatic reduction in sample size at day 24? (from 47 to 22 pig samples in just two days?)

A subset of 12 CHCF1 RS and 12 CHCF1 RR pigs were selected for in-vivo testing of susceptibility to ETEC F4. The number of animals exposed to experimentally induced disease should generally be kept at a minimum for welfare reasons. For details on study design please refer to Fig 1. Lines 93-95 and 111-115.

L193-199: please add the model equations: it is fine to describe them, but the formal notation would help better understand important details (e.g. covariance structure, nested effects etc.)

We appreciate the suggestion to include formal model equations. However, we believe that adding these equations may not enhance the readability or accessibility of the statistical methods section. The key details regarding fixed and random effects, as well as other important aspects of the models, are thoroughly described in the text for each analysis performed. We aim to ensure that the section remains clear and comprehensible to a broad audience.

For the linear mixed models concerning alpha diversity measures the model equations (lines 192-198), where CHCF1 genotype is as fixed effect and litter as random effect can be described as:

$$y_{ij}=\beta_{0}+\beta_{1}\text{CHCF1}_{i}+u_{j}+\epsilon_{ij}$$

Where, *y_ij_* is the response variable for the (i)-th observation in the (j)-th litter. β_0_ is the intercept. β_1_ is the fixed effect of the CHCF1 genotype. u_j_ is the random effect of the (j)-th litter. ϵ_ij_ is the residual error.

And for the longitudinal analysis with CHCF1 genotype and age (scaled) as fixed effects and with pig and sow as random effects:

2) $y_{ijk}=\beta_{0}+\beta_{1}\text{CHCF1}+\beta_{2}\text{Age}_{k}+u_{j}+v_{k}+\epsilon_{ijk}$

Where, *y_ijk_* is the response variable for the (i)-th observation in the (j)-th pig and (k)-th sow. β_0_ is the intercept. β_1_ is the fixed effect of the CHCF1 genotype. β_2_ is the fixed effect of the scaled age. u_j_ is the random effect of the (j)-th pig. v_k_ is the random effect of the (k)-th sow. ϵ_ijk_ is the residual error.

However, for the PERMANOVA analysis and ANCOMBC differential abundance analysis, the model equations are more comprehensive and involve more steps (ANCOMBC-2). We believe the mathematical details and procedures are best captured by referring to the original papers and R package descriptions. Readers can find all relevant information on fixed and random effects included in our models described in the text.

L205: what is the betadisper() function? Please describe the methods, not only the operational implementations

We have added a reference to the original article developing and describing the test by Marti Anderson, line 205. The test checks for multivariate homogeneity of group dispersions, in other words if one or more groups have sample data that are significantly more scattered around the centroid than others. This is important as variability in dispersions between groups can potentially lead to false detection of a differences in means in PERMANOVA analysis.

L212: Prevalence cut was set to 10%: was this for the differential abundance analysis of OTUs between groups? 10% seems to be a high threshold to remove OTUs (at genus level?)

Yes, for assessing differential abundance between groups the prevalence cut was set to 10% in OTU and genus level analysis. In ANCOMBC-2, a prevalence cut of 10% is the default setting. We chose this threshold to focus on differences in OTUs/genera shared by at least 10% of the pigs. We believe this is the most appropriate approach focusing on differences that are more likely to be biologically meaningful and not driven by rare occurrences. Furthermore, a lower prevalence cut could potentially obscure true differences because it would involve having to p-adjust for more OTUs/genera being analyzed.

Following your question, we explored what would happen if prevalence cut was set to 5% for genus level analysis. The results led to the same conclusion, that there were no differential abundant genera between the two groups at any of the three timepoints.

Fig. 1: "swabs taken at post-natal day (PND) 8, 22 for pre-weaning comparison" This sentence is a little unclear: taken at days 8 and 22?

Changed to “swabs taken at post-natal day (PND) 8 and 22 for pre-weaning comparison” line 117-118.
