# Supplementary material for: The early-life fecal microbiota is associated with litter of origin but not with susceptibility to ETEC F4ab-mediated post-weaning diarrhea in CHCF1 genotyped pigs
Source: PLoS One. 2025 May 29;20(5):e0323875. doi: 10.1371/journal.pone.0323875 (PMC12121822; doi:10.1371/journal.pone.0323875)
Supplement: Peer review history

# Author comment: Author response to Decision Letter 0

**DOI:** 10.1371/journal.pone.0323875.r001

22 Jul 2024

# Peer review: Decision Letter 0

**DOI:** 10.1371/journal.pone.0323875.r002

**Authors:** Mohamed O Ahmed

31 Oct 2024

PONE-D-24-30472The early-life fecal microbiota is associated with litter of origin but not with susceptibility to ETEC F4ab-mediated post-weaning diarrhea in CHCF1 genotyped pigsPLOS ONE

Dear Dr. Rydal,

Thank you for submitting your manuscript to PLOS ONE. After careful consideration, we feel that it has merit but does not fully meet PLOS ONE’s publication criteria as it currently stands. Therefore, we invite you to submit a revised version of the manuscript that addresses the points raised during the review process.

Please submit your revised manuscript by Dec 15 2024 11:59PM. If you will need more time than this to complete your revisions, please reply to this message or contact the journal office at plosone@plos.org . When you're ready to submit your revision, log on to https://www.editorialmanager.com/pone/ and select the 'Submissions Needing Revision' folder to locate your manuscript file.

Please include the following items when submitting your revised manuscript:

- A rebuttal letter that responds to each point raised by the academic editor and reviewer(s). You should upload this letter as a separate file labeled 'Response to Reviewers'.
- A marked-up copy of your manuscript that highlights changes made to the original version. You should upload this as a separate file labeled 'Revised Manuscript with Track Changes'.
- An unmarked version of your revised paper without tracked changes. You should upload this as a separate file labeled 'Manuscript'.

If you would like to make changes to your financial disclosure, please include your updated statement in your cover letter. Guidelines for resubmitting your figure files are available below the reviewer comments at the end of this letter.

If applicable, we recommend that you deposit your laboratory protocols in protocols.io to enhance the reproducibility of your results. Protocols.io assigns your protocol its own identifier (DOI) so that it can be cited independently in the future. For instructions see: https://journals.plos.org/plosone/s/submission-guidelines#loc-laboratory-protocols . Additionally, PLOS ONE offers an option for publishing peer-reviewed Lab Protocol articles, which describe protocols hosted on protocols.io. Read more information on sharing protocols at https://plos.org/protocols?utm_medium=editorial-email&utm_source=authorletters&utm_campaign=protocols .

We look forward to receiving your revised manuscript.

Kind regards,

Mohamed O Ahmed, Ph.D

Academic Editor

PLOS ONE

Journal requirements: When submitting your revision, we need you to address these additional requirements. 1. Please ensure that your manuscript meets PLOS ONE's style requirements, including those for file naming. The PLOS ONE style templates can be found at https://journals.plos.org/plosone/s/file?id=wjVg/PLOSOne_formatting_sample_main_body.pdf and https://journals.plos.org/plosone/s/file?id=ba62/PLOSOne_formatting_sample_title_authors_affiliations.pdf 2. To comply with PLOS ONE submissions requirements, in your Methods section, please provide additional information regarding the experiments involving animals and ensure you have included details on methods of anesthesia and/or analgesia, and efforts to alleviate suffering. 3. Please include captions for your all Supporting Information files at the end of your manuscript, and update any in-text citations to match accordingly. Please see our Supporting Information guidelines for more information: http://journals.plos.org/plosone/s/supporting-information. 

[Note: HTML markup is below. Please do not edit.]

Reviewers' comments:

Reviewer's Responses to Questions

**Comments to the Author**

1. Is the manuscript technically sound, and do the data support the conclusions?

The manuscript must describe a technically sound piece of scientific research with data that supports the conclusions. Experiments must have been conducted rigorously, with appropriate controls, replication, and sample sizes. The conclusions must be drawn appropriately based on the data presented. 

Reviewer #1: No

Reviewer #2: Yes

**********

2. Has the statistical analysis been performed appropriately and rigorously? 

Reviewer #1: No

Reviewer #2: Yes

**********

3. Have the authors made all data underlying the findings in their manuscript fully available?

The PLOS Data policy requires authors to make all data underlying the findings described in their manuscript fully available without restriction, with rare exception (please refer to the Data Availability Statement in the manuscript PDF file). The data should be provided as part of the manuscript or its supporting information, or deposited to a public repository. For example, in addition to summary statistics, the data points behind means, medians and variance measures should be available. If there are restrictions on publicly sharing data—e.g. participant privacy or use of data from a third party—those must be specified.

Reviewer #1: Yes

Reviewer #2: Yes

**********

4. Is the manuscript presented in an intelligible fashion and written in standard English?

PLOS ONE does not copyedit accepted manuscripts, so the language in submitted articles must be clear, correct, and unambiguous. Any typographical or grammatical errors should be corrected at revision, so please note any specific errors here.

Reviewer #1: No

Reviewer #2: Yes

**********

5. Review Comments to the Author

Please use the space provided to explain your answers to the questions above. You may also include additional comments for the author, including concerns about dual publication, research ethics, or publication ethics. (Please upload your review as an attachment if it exceeds 20,000 characters)

Reviewer #1: The manuscript attempts to describe an interesting investigation of potential connection of early-life microbiota and susceptibility to ETEC. However, the manuscript is lacking in many regards.

Lines 91-111: The Experimental design is poorly described. This sounds like a 2x2 factorial, so it should be declared that way.

Line 91: replace "randomized" with "selected"

Line 93: How many pigs were selected from each sow? Were the sows profiled?

Lines 93-94: How were pigs identified as RS or RR?

Lines 94-95: This seems out of order.

Line 97: Where and how were pigs housed?

Lines 100-101: This does not belong here, rather Discussion.

Lines 101-102: This only accounts for 24 of 48 pigs.

Line 106: What were the treatment groups exactly?

Line 108: Were all the pigs challenged with ETEC?

Line 119: Provide at least some details of the methods.

Line 122: insert "Quality control" before abbreviation

Lines 123-124: Was it <10000 or <8000?

Line 128: Was the protocol from a published study or the manufacturer?

Line 138: Is the description of the custom protocol the text that follows?

Line 182: What were the experimental groups? Were all the factors and interactions considered?

Line 186: insert "Operational taxonomic unit" before abbreviation

Line 199: Define the groups being considered.

Line 222: What were the responses of the pigs not challenged with ETEC?

Line 236: What was the comparison?

Line 242: What was the comparison?

Lines 307-end: The Discussion must be rewritten considering the updated statistical analysis.

Reviewer #2: The manuscript delivers a good study with well-executed experiments and unambiguous conclusions. However, it would benefit from putting a stronger emphasis on non-significant findings in the results section and simplifying some of the technical terminology in the methods to improve accessibility. Furthermore, focusing on the implications of the litter effect in the discussion would increase the significance of the findings. Overall, minimal rivisions are suggested to improve clarity and presentation.

**********

6. PLOS authors have the option to publish the peer review history of their article (what does this mean? ). If published, this will include your full peer review and any attached files.

If you choose “no”, your identity will remain anonymous but your review may still be made public.

**Do you want your identity to be public for this peer review?** For information about this choice, including consent withdrawal, please see our Privacy Policy .

Reviewer #1: **Yes: ** Ryan Samuel

Reviewer #2: No

**********

[NOTE: If reviewer comments were submitted as an attachment file, they will be attached to this email and accessible via the submission site. Please log into your account, locate the manuscript record, and check for the action link "View Attachments". If this link does not appear, there are no attachment files.]

While revising your submission, please upload your figure files to the Preflight Analysis and Conversion Engine (PACE) digital diagnostic tool, https://pacev2.apexcovantage.com/ . PACE helps ensure that figures meet PLOS requirements. To use PACE, you must first register as a user. Registration is free. Then, login and navigate to the UPLOAD tab, where you will find detailed instructions on how to use the tool. If you encounter any issues or have any questions when using PACE, please email PLOS at figures@plos.org . Please note that Supporting Information files do not need this step.

Attachment Submitted filename: review comments.pdf

# Author comment: Author response to Decision Letter 1

**DOI:** 10.1371/journal.pone.0323875.r003

12 Dec 2024

Reviewer 1:

Dear reviewer 1,

Thank you for your valuable time and well-considered input. Please find our elaborated response below in red.

Lines 91-111: The Experimental design is poorly described. This sounds like a 2x2 factorial, so it should be declared that way.

The study was a longitudinal, observational study with stratified randomization. We did this randomization technique to ensure that the CHCF1 RS and CHCF1 RR groups were balanced in terms of numbers of pigs with susceptible and resistant genotypes. The subset of pigs that were followed after weaning prior to an ETEC F4 challenged were randomized stratified by both CHCF1 genotype and body weight to ensure that the groups were also balanced in terms of body weight at the experimental facility (Fig 1, SI 1 Fig).

More details can now be found in the manuscript, lines 104-122.

Line 91: replace "randomized" with "selected"

Updated line 106: “randomized in blocks”

We believe “randomized” is the more correct term, as the study population was indeed chosen by randomizing available pigs and not merely “selected”, which we believe can be misread as convenience sampling.

Line 93: How many pigs were selected from each sow? Were the sows profiled?

Ranging from 5-9 pigs (Fig 5). Information added to “Animals” section in the methods, lines 87-88.

The sows were not genotyped.

Lines 93-94: How were pigs identified as RS or RR?

Pigs were identified as RS or RR based on the CHCF1 allele marker, where:

CHCF1 (resistant allele = C)

ACATAGAGACTTTCTAGCAAAGTTGCTAGGAGAGCAAATCTTAAAAGTTCT[C/A]AAGG

CGGAGTTCCCATTGTGGCACAGTGGAAATGGATCTGACTGGGAAC

More information on the genotyping can be found in:

https://doi.org/10.1016/j.vetmic.2023.109771.

Lines 94-95: This seems out of order.

We have updated the sentence to clarify what was compared. Updated line 109-110:

“The fecal microbiotas of the 24 CHCF1 RS and 24 CHCF1 RR pigs were compared at early lactation (PND 8) and at late lactation (PND 22).”

Line 97: Where and how were pigs housed?

At the experimental facility, the 12 CHCF1 RS and 12 CHCF1 were housed in two separate rooms with one pen per room. We did this and used strict hygiene standards to avoid cross contamination of the challenge strain between groups. We have updated “Animals” section of the methods for clarity, lines 95-96.

Details on the housing conditions at the experimental facility has been described in detail in the paper describing the infection trial .

Lines 100-101: This does not belong here, rather Discussion.

We have removed this information from the experimental design section.

Lines 101-102: This only accounts for 24 of 48 pigs.

We were referring only to the subpopulation of pigs that were part of the subsequent ETEC challenge trial. The information has been removed from the experimental design section, as it already appears in the discussion, lines 319-321.

Line 106: What were the treatment groups exactly?

We followed a group of CHCF1 RS pigs and a group of CHCF1 RR pigs from the same herd and sows that had the same conditions in terms of feeding, housing and management. They only differed in CHCF1 genotype.

A visualization of the microbiota study design is available in Fig 1. Briefly, we first looked at differences in fecal microbiota composition between 24 CHCF1 RS and 24 RR pigs at two pre-weaning timepoints at the herd (PND 8 and PND 22). Then a subset of 12 CHCF1 RS and 12 CHCF1 RR pigs were randomized for an experimental challenge trial, where we looked at fecal microbiota difference 1 day after weaning, just before an experimental challenge with ETEC F4ab. In the current microbiota study, we only followed the 12 CHCF1 RS and 12 CHCF1 RR pigs till PND 24 (1 day after weaning) at the experimental facility.

The subsequent infection trial included the two groups described above: CHCF1 RS (n=12) and CHCF1 RR (n=12). The two groups received the same feed and ETEC F4ab challenge in terms of strain, dose and timing. They differed only in genotype. The response to ETEC inoculation in terms of diarrhea development and clinical condition has been described in a paper describing the infection trial1. In the paper on the infection trial1, you can find that all the 12 CHCF1 RS developed ETEC F4ab –mediated diarrhea whereas none of the 12 CHCF1 RR pigs did. This is mentioned in the discussion (lines 319-321) as it shows that pigs in our microbiota study were not just genetically susceptible/resistant, but were also confirmed to be clinically susceptible/resistant to ETEC F4ab.

Line 108: Were all the pigs challenged with ETEC?

Only the subset of 12 CHCF1 RS and 12 CHCF1 RR pigs that were included in the experimental infection trial (Fig 1), lines 111-115.

Line 119: Provide at least some details of the methods.

The method on Oxford nanopore long-read sequencing of the bacterial 16S rRNA gene have been updated for improved accessibility for readers. Lines 127-179

Line 122: insert "Quality control" before abbreviation

Same as above.

Lines 123-124: Was it <10000 or <8000?

Below 8000.

Line 128: Was the protocol from a published study or the manufacturer?

Manufacturer.

https://www.mpbio.com/us/116560000-fastdna-spin-kit-for-soil-samp-cf?srsltid=AfmBOoqc8wzg4tP4fxydJIsJbPlR04qCFuVlO4e90CeWMIdlNiGP95g7

Line 138: Is the description of the custom protocol the text that follows?

Yes.

Line 182: What were the experimental groups? Were all the factors and interactions considered?

To clarify experimental groups are added to the statistical method, lines 182-184.

With great effort, the study was designed to minimize potential confounding factors (lines 85-96) thereby making it possible to simplify analysis.

For example,

1) By rearing pigs with their mother sow in the pen that they were born in at the farrowing unit. Thereby, we avoided cross-fostering which was a potential confounder for the fecal microbiota.

2) At birth, only pigs weighing between 1-2 kg were included. Thereby we avoided “outlier pigs” with low or very high birthweight. Now described in line 88.

3) We only included female pigs to avoid potential sex differences.

4) Pigs had the same housing, management and feeding conditions.

5) Pigs that were randomized for the infection trial were stratified by weaning weight to ensure that the same weight classes were equally represented between the CHCF1 RS and CHCF1 RR group.

6) We did not include litters from gilts.

Given the design of the study, it was appropriate to account for the random effect of litter (littermates cannot be seen as completely independent in terms of microbiota composition) in addition to the explanatory variable of interest (CHCF1 genotype) in analysis of alpha and beta diversity as well as in differential abundance analysis.

Line 186: insert "Operational taxonomic unit" before abbreviation

Now appears for the first time in in lines 177-178.

Line 199: Define the groups being considered.

“Differences in beta diversity between groups were assessed using Canberra and binary jaccard distance matrices and analyzed with PERMANOVA at 999 permutations.”

Has been updated to

“Differences in beta diversity between CHCF1 RS and CHCF1 RR groups were assessed using Canberra and binary jaccard distance matrices and analyzed with PERMANOVA at 999 permutations.”

Lines 200-202

Line 222: What were the responses of the pigs not challenged with ETEC?

All the 12 CHCF1 RS and 12 CHCF1 RR pigs that were included in the infection trial were challenged with the same dose of an ETEC F4ab strain. Since all were challenged, we cannot say anything about responses of pigs not challenged with ETEC.

The additional 12 CHCF1 RS and 12 CHCF1 RR that remained at the supplying herd were not continuously followed in the nursery.

Line 236: What was the comparison?

CHCF1 RS pigs were compared with CHCF1 RR pigs across time.

Updated for clarification, line 237:

“Analysis for difference between CHCF1 RS and RR genotypes across timepoints…”

Line 242: What was the comparison?

All pig samples, irrespective of genotype, are shown in boxplots at the three different timepoints (PND 8, PND 22 and PND 24) to visualize the development in alpha diversity across time (Fig 3).

Lines 307-end: The Discussion must be rewritten considering the updated statistical analysis.

Thank you for the comment. Given our explanation to your previous question “Line 182: What were the experimental groups? Were all the factors and interactions considered?”, we do not see a need for reanalyzing the data.

Reviewer 2:

Dear reviewer 2,

Thank you for your important insights and valuable time. Please find our elaborated response below in red.

The manuscript delivers a good study with well-executed experiments and unambiguous conclusions. However, it would benefit from putting a stronger emphasis on non-significant findings in the results section…

We agree with your point and have updated the results section to further increase transparency to the reader. Lines 242-254.

…and simplifying some of the technical terminology in the methods to improve accessibility.

We have simplified technical terminology in the part of the methods describing Oxford nanopore long-read sequencing of the bacterial 16S rRNA gene to improve reader accessibility. Lines 127-179

Furthermore, focusing on the implications of the litter effect in the discussion would increase the significance of the findings.

We have added thoughts on implications of the litter effect to the discussion, lines 369-377.

Editor comments:

Dear Dr. Mohamed O Ahmed,

Thank you for your important comments and time spend to help improve our manuscript. Please find our elaborated response below in red.

Areas for Improvement:

Line 22-24: The genetic vulnerability to ETEC F4ab/ac statement in the introduction would

benefit from further context regarding the significance of these discoveries in larger pig

production systems or possible human uses. For clarity, think about going into more detail.

We have elaborated in the introduction of the abstract for further clarity. Lines 20-26.

Line 25-30: The study population and timing are clearly described, yet there may have been

a more seamless transition from discussing genetic susceptibility to outlining the

experimental challenge. You can briefly discuss the significance of PND 8, PND 22, and

PND 24 time points for the study.

We have elaborated the method part of the abstract to address your point. Lines 27-35.

Line 117-177: There is a lot of technical jargon in this section. Think about elucidating

terminology like “filtlong” and “minimap2” in further detail or simplifying them for readers

who might not be familiar with bioinformatic tools. Accessibility and readability would both

be enhanced by this.

We have simplified technical terminology in the part of the methods describing Oxford nanopore long-read sequencing of the bacterial 16S rRNA gene to improve readability. Lines 127-179

Line 220-231: The explanation of the alpha diversity analysis results is clear, yet it would be

beneficial to draw attention to the statistical insignificance of several measurements, such

as the p-values of 0.05 and 0.4 for the Shannon and Simpson indices, respectively. This

keeps results that are close to significance but fall short of the cutoV from being

overinterpreted.

We have elaborated on the non-significant results in alpha diversity to further increase transparency to the reader. Lines 242-254.

Line 310-317: When discussing the higher alpha diversity in CHCF1 susceptible pigs, the

potential explanations are well-presented, but further clarification about the statistical

insignificance of E. coli abundance (line 316-317) should be highlighted. Emphasize that

these trends were not statistically significant to avoid confusion.

We have updated the discussion to emphasize that the numbers and abundances of E. coli were not statistically different. Lines 331-334.

Line 221-239: You mention trends in Simpson indices and Shannon diversity in the

discussion of alpha diversity. When using the term "tendencies" in scientific reporting, be

sure to define it precisely. While making a suggestion of a potential trend, you might clearly

say that these numbers did not satisfy the significance threshold.

Thank you for the comment. We used the term “tendencies” if p-values were below 0.1 but failed to satisfy the significant threshold set to below 0.05. We defined p-values below 0.1 as tendencies in the statistical method section, line 182.

Line 325-327: Although the explanation of the possible roles played by various strains of E.

coli is informative, it might be more precise. Even if there were higher OTU numbers, the

diVerences were not statistically significant, thus you could still have a compelling

hypothesis for further study.

We agree with your comment and hope that our updated discussion helps to clarify this point, lines 339-340.

Attachment Submitted filename: Response to Reviewers.docx

# Peer review: Decision Letter 1

**DOI:** 10.1371/journal.pone.0323875.r004

**Authors:** Mohamed O Ahmed

23 Jan 2025

PONE-D-24-30472R1The early-life fecal microbiota is associated with litter of origin but not with susceptibility to ETEC F4ab-mediated post-weaning diarrhea in CHCF1 genotyped pigsPLOS ONE

Dear Dr. Rydal,

Thank you for submitting your manuscript to PLOS ONE. After careful consideration, we feel that it has merit but does not fully meet PLOS ONE’s publication criteria as it currently stands. Therefore, we invite you to submit a revised version of the manuscript that addresses the points raised during the review process.

Please submit your revised manuscript by Mar 09 2025 11:59PM. If you will need more time than this to complete your revisions, please reply to this message or contact the journal office at plosone@plos.org . When you're ready to submit your revision, log on to https://www.editorialmanager.com/pone/ and select the 'Submissions Needing Revision' folder to locate your manuscript file.

Please include the following items when submitting your revised manuscript:

- A rebuttal letter that responds to each point raised by the academic editor and reviewer(s). You should upload this letter as a separate file labeled 'Response to Reviewers'.
- A marked-up copy of your manuscript that highlights changes made to the original version. You should upload this as a separate file labeled 'Revised Manuscript with Track Changes'.
- An unmarked version of your revised paper without tracked changes. You should upload this as a separate file labeled 'Manuscript'.

If you would like to make changes to your financial disclosure, please include your updated statement in your cover letter. Guidelines for resubmitting your figure files are available below the reviewer comments at the end of this letter.

If applicable, we recommend that you deposit your laboratory protocols in protocols.io to enhance the reproducibility of your results. Protocols.io assigns your protocol its own identifier (DOI) so that it can be cited independently in the future. For instructions see: https://journals.plos.org/plosone/s/submission-guidelines#loc-laboratory-protocols . Additionally, PLOS ONE offers an option for publishing peer-reviewed Lab Protocol articles, which describe protocols hosted on protocols.io. Read more information on sharing protocols at https://plos.org/protocols?utm_medium=editorial-email&utm_source=authorletters&utm_campaign=protocols .

We look forward to receiving your revised manuscript.

Kind regards,

Mohamed O Ahmed, Ph.D

Academic Editor

PLOS ONE

[Note: HTML markup is below. Please do not edit.]

Reviewers' comments:

Reviewer's Responses to Questions

**Comments to the Author**

1. If the authors have adequately addressed your comments raised in a previous round of review and you feel that this manuscript is now acceptable for publication, you may indicate that here to bypass the “Comments to the Author” section, enter your conflict of interest statement in the “Confidential to Editor” section, and submit your "Accept" recommendation.

Reviewer #1: All comments have been addressed

Reviewer #3: (No Response)

**********

2. Is the manuscript technically sound, and do the data support the conclusions?

The manuscript must describe a technically sound piece of scientific research with data that supports the conclusions. Experiments must have been conducted rigorously, with appropriate controls, replication, and sample sizes. The conclusions must be drawn appropriately based on the data presented. 

Reviewer #1: Yes

Reviewer #3: Yes

**********

3. Has the statistical analysis been performed appropriately and rigorously? 

Reviewer #1: Yes

Reviewer #3: Yes

**********

4. Have the authors made all data underlying the findings in their manuscript fully available?

The PLOS Data policy requires authors to make all data underlying the findings described in their manuscript fully available without restriction, with rare exception (please refer to the Data Availability Statement in the manuscript PDF file). The data should be provided as part of the manuscript or its supporting information, or deposited to a public repository. For example, in addition to summary statistics, the data points behind means, medians and variance measures should be available. If there are restrictions on publicly sharing data—e.g. participant privacy or use of data from a third party—those must be specified.

Reviewer #1: Yes

Reviewer #3: Yes

**********

5. Is the manuscript presented in an intelligible fashion and written in standard English?

PLOS ONE does not copyedit accepted manuscripts, so the language in submitted articles must be clear, correct, and unambiguous. Any typographical or grammatical errors should be corrected at revision, so please note any specific errors here.

Reviewer #1: Yes

Reviewer #3: Yes

**********

6. Review Comments to the Author

Please use the space provided to explain your answers to the questions above. You may also include additional comments for the author, including concerns about dual publication, research ethics, or publication ethics. (Please upload your review as an attachment if it exceeds 20,000 characters)

Reviewer #1: (No Response)

Reviewer #3: Title : The early-life fecal microbiota is associated with litter of origin but not with susceptibility to ETEC F4ab-mediated post-weaning diarrhea in CHCF1 genotyped pigs

- I believe that this 2022 article in PlosONE on "Gut microbiome modifications over time when removing in-feed antibiotics from the prophylaxis of post-weaning diarrhea in piglets" can be beneficial for the Introduction or Discussion of your work (Cremonesi et al. 2022, PLoS ONE 17(3): e0262199. https://doi.org/10.1371/journal.pone.0262199)

- it would be much better the place figures and tables within the text, otherwise the revision process becomes more complicated, and the reading experience is inconvenienced

- figures are at low resolution hence not easy to read/assess

Introduction

------------

- I suggest that you spell out the abbreviations -e.g. ETEC, PWD- the first time you introduce them in the main manuscript; not all readers will necessarily read the abstract as the first thing

L99: what are humane endpoints? Please be a little more specific

Methods

-------

L176-177: "an abundance count of the database entries mapped to, combined with their database-derived taxonomic assignment" : this sentence is quite convoluted and not easy to understand, please rephrase

L181-182: you did not use any strategy to check for potentially spurious results? (p-values may come up as significant merely by chance)

L185: mapped reads

L186: why this dramatic reduction in sample size at day 24? (from 47 to 22 pig samples in just two days?)

L193-199: please add the model equations: it is fine to describe them, but the formal notation would help better understand important details (e.g. covariance structure, nested effects etc.)

L205: what is the betadisper() function? Please describe the methods, not only the operational implementations

L212: Prevalence cut was set to 10%: was this for the differential abundance analysis of OTUs between groups? 10% seems to be a high threshold to remove OTUs (at genus level?)

Fig. 1: "swabs taken at post-natal day (PND) 8, 22 for pre-weaning comparison" This sentence is a little unclear: taken at days 8 and 22?

**********

7. PLOS authors have the option to publish the peer review history of their article (what does this mean? ). If published, this will include your full peer review and any attached files.

If you choose “no”, your identity will remain anonymous but your review may still be made public.

**Do you want your identity to be public for this peer review?** For information about this choice, including consent withdrawal, please see our Privacy Policy .

Reviewer #1: No

Reviewer #3: No

**********

[NOTE: If reviewer comments were submitted as an attachment file, they will be attached to this email and accessible via the submission site. Please log into your account, locate the manuscript record, and check for the action link "View Attachments". If this link does not appear, there are no attachment files.]

While revising your submission, please upload your figure files to the Preflight Analysis and Conversion Engine (PACE) digital diagnostic tool, https://pacev2.apexcovantage.com/ . PACE helps ensure that figures meet PLOS requirements. To use PACE, you must first register as a user. Registration is free. Then, login and navigate to the UPLOAD tab, where you will find detailed instructions on how to use the tool. If you encounter any issues or have any questions when using PACE, please email PLOS at figures@plos.org . Please note that Supporting Information files do not need this step.

# Author comment: Author response to Decision Letter 2

**DOI:** 10.1371/journal.pone.0323875.r005

13 Feb 2025

Response to Reviewers

Dear reviewer 3,

Thank you for your well-considered comments and time spend to help improve our manuscript. Please find our elaborate response below in red.

- I believe that this 2022 article in PlosONE on "Gut microbiome modifications over time when removing in-feed antibiotics from the prophylaxis of post-weaning diarrhea in piglets" can be beneficial for the Introduction or Discussion of your work (Cremonesi et al. 2022, PLoS ONE 17(3): e0262199. https://doi.org/10.1371/journal.pone.0262199)

Thank you for the reference, added line 362.

- it would be much better the place figures and tables within the text, otherwise the revision process becomes more complicated, and the reading experience is inconvenienced

Thank you for the comment and sorry for the inconvenience, however we must follow journal submission guidelines that state under “Figures”:

“Do not include figures in the main manuscript file. Each figure must be prepared and submitted as an individual file.”

https://journals.plos.org/plosone/s/submission-guidelines

- figures are at low resolution hence not easy to read/assess

We have reuploaded figures with increased resolution to enhance reading experience.

Introduction

------------

- I suggest that you spell out the abbreviations -e.g. ETEC, PWD- the first time you introduce them in the main manuscript; not all readers will necessarily read the abstract as the first thing

Corrected, line 51.

L99: what are humane endpoints? Please be a little more specific

Thank you for the comment.

We described humane endpoints in detail in our paper on the infection trial:

“The pigs were scored clinically twice a day and assessment included wet perianal fecal staining, body condition score, signs of dehydration (rough hair coat, sunken eyes and flanks, skin turgor by pinch skinfold test), cyanosis and affected mental status (lay down a lot, had to be encouraged to move). Temperature and body weight was measured once daily and on indication. In addition, the pigs were clinically assessed every 3 h and on indication after inoculation until the end of the study period according to Additional file 1. In case of a clinical score 3, pigs would be monitored regularly and if they did not improve within 3 h they would be euthanized for humane reasons.”

Quote. Rydal MP, Jørgensen CB, Gambino M, Poulsen LL, Nielsen JP. Complete association between CHCF1 genotype and enterotoxigenic Escherichia coli F4ab-associated post-weaning diarrhea in a pig challenge trial. Vet Microbiol. 2023 Jul;282:109771. doi: 10.1016/j.vetmic.2023.109771. Epub 2023 May 3. PMID: 37150059.

We reference to this article in line 103 so readers can find all information on humane endpoints that has already been described and published.

Methods

-------

L176-177: "an abundance count of the database entries mapped to, combined with their database-derived taxonomic assignment" : this sentence is quite convoluted and not easy to understand, please rephrase

Rephrased to:

“Taken together, this produced a so-called observed taxonomic unit (OTU) table.”

Line 176.

L181-182: you did not use any strategy to check for potentially spurious results? (p-values may come up as significant merely by chance)

We used the Holm-Bonferonni method to adjust p-values. Line 212-213 and 218.

L185: mapped reads

Corrected, line 184.

L186: why this dramatic reduction in sample size at day 24? (from 47 to 22 pig samples in just two days?)

A subset of 12 CHCF1 RS and 12 CHCF1 RR pigs were selected for in-vivo testing of susceptibility to ETEC F4. The number of animals exposed to experimentally induced disease should generally be kept at a minimum for welfare reasons. For details on study design please refer to Fig 1. Lines 93-95 and 111-115.

L193-199: please add the model equations: it is fine to describe them, but the formal notation would help better understand important details (e.g. covariance structure, nested effects etc.)

We appreciate the suggestion to include formal model equations. However, we believe that adding these equations may not enhance the readability or accessibility of the statistical methods section. The key details regarding fixed and random effects, as well as other important aspects of the models, are thoroughly described in the text for each analysis performed. We aim to ensure that the section remains clear and comprehensible to a broad audience.

For the linear mixed models concerning alpha diversity measures the model equations (lines 192-198), where CHCF1 genotype is as fixed effect and litter as random effect can be described as:

y_ij=β_0+β_1 〖"CHCF1" 〗_i+u_j+ϵ_ij

Where, yij is the response variable for the (i)-th observation in the (j)-th litter. β0 is the intercept. β1 is the fixed effect of the CHCF1 genotype. uj is the random effect of the (j)-th litter. ϵij is the residual error.

And for the longitudinal analysis with CHCF1 genotype and age (scaled) as fixed effects and with pig and sow as random effects:

2) y_ijk=β_0+β_1 "CHCF1"+β_2 〖"Age" 〗_k+u_j+v_k+ϵ_ijk

Where, yijk is the response variable for the (i)-th observation in the (j)-th pig and (k)-th sow. β0 is the intercept. β1 is the fixed effect of the CHCF1 genotype. β2 is the fixed effect of the scaled age. uj is the random effect of the (j)-th pig. vk is the random effect of the (k)-th sow. ϵijk is the residual error.

However, for the PERMANOVA analysis and ANCOMBC differential abundance analysis, the model equations are more comprehensive and involve more steps (ANCOMBC-2). We believe the mathematical details and procedures are best captured by referring to the original papers and R package descriptions. Readers can find all relevant information on fixed and random effects included in our models described in the text.

L205: what is the betadisper() function? Please describe the methods, not only the operational implementations

We have added a reference to the original article developing and describing the test by Marti Anderson, line 205. The test checks for multivariate homogeneity of group dispersions, in other words if one or more groups have sample data that are significantly more scattered around the centroid than others. This is important as variability in dispersions between groups can potentially lead to false detection of a differences in means in PERMANOVA analysis.

L212: Prevalence cut was set to 10%: was this for the differential abundance analysis of OTUs between groups? 10% seems to be a high threshold to remove OTUs (at genus level?)

Yes, for assessing differential abundance between groups the prevalence cut was set to 10% in OTU and genus level analysis. In ANCOMBC-2, a prevalence cut of 10% is the default setting. We chose this threshold to focus on differences in OTUs/genera shared by at least 10% of the pigs. We believe this is the most appropriate approach focusing on differences that are more likely to be biologically meaningful and not driven by rare occurrences. Furthermore, a lower prevalence cut could potentially obscure true differences because it would involve having to p-adjust for more OTUs/genera being analyzed.

Following your question, we explored what would happen if prevalence cut was set to 5% for genus level analysis. The results led to the same conclusion, that there were no differential abundant genera between the two groups at any of the three timepoints.

Fig. 1: "swabs taken at post-natal day (PND) 8, 22 for pre-weaning comparison" This sentence is a little unclear: taken at days 8 and 22?

Changed to “swabs taken at post-natal day (PND) 8 and 22 for pre-weaning comparison” line 117-118.

Attachment Submitted filename: Response_to_Reviewers_auresp_2.docx

# Peer review: Decision Letter 2

**DOI:** 10.1371/journal.pone.0323875.r006

**Authors:** Mohamed O Ahmed

16 Apr 2025

The early-life fecal microbiota is associated with litter of origin but not with susceptibility to ETEC F4ab-mediated post-weaning diarrhea in CHCF1 genotyped pigs

PONE-D-24-30472R2

Dear Dr. Rydal,

We’re pleased to inform you that your manuscript has been judged scientifically suitable for publication and will be formally accepted for publication once it meets all outstanding technical requirements.

Within one week, you’ll receive an e-mail detailing the required amendments. When these have been addressed, you’ll receive a formal acceptance letter and your manuscript will be scheduled for publication.

An invoice will be generated when your article is formally accepted. Please note, if your institution has a publishing partnership with PLOS and your article meets the relevant criteria, all or part of your publication costs will be covered. Please make sure your user information is up-to-date by logging into Editorial Manager at Editorial Manager®  and clicking the ‘Update My Information' link at the top of the page. If you have any questions relating to publication charges, please contact our Author Billing department directly at authorbilling@plos.org.

If your institution or institutions have a press office, please notify them about your upcoming paper to help maximize its impact. If they’ll be preparing press materials, please inform our press team as soon as possible -- no later than 48 hours after receiving the formal acceptance. Your manuscript will remain under strict press embargo until 2 pm Eastern Time on the date of publication. For more information, please contact onepress@plos.org.

Kind regards,

Mohamed O Ahmed, Ph.D

Academic Editor

PLOS ONE

# Editor report: Acceptance letter

**DOI:** 10.1371/journal.pone.0323875.r007

**Authors:** Mohamed O Ahmed

PONE-D-24-30472R2

PLOS ONE

Dear Dr. Rydal,

I'm pleased to inform you that your manuscript has been deemed suitable for publication in PLOS ONE. Congratulations! Your manuscript is now being handed over to our production team.

At this stage, our production department will prepare your paper for publication. This includes ensuring the following:

* All references, tables, and figures are properly cited

* All relevant supporting information is included in the manuscript submission,

* There are no issues that prevent the paper from being properly typeset

You will receive further instructions from the production team, including instructions on how to review your proof when it is ready. Please keep in mind that we are working through a large volume of accepted articles, so please give us a few days to review your paper and let you know the next and final steps.

Lastly, if your institution or institutions have a press office, please let them know about your upcoming paper now to help maximize its impact. If they'll be preparing press materials, please inform our press team within the next 48 hours. Your manuscript will remain under strict press embargo until 2 pm Eastern Time on the date of publication. For more information, please contact onepress@plos.org.

If we can help with anything else, please email us at customercare@plos.org.

Thank you for submitting your work to PLOS ONE and supporting open access.

Kind regards,

PLOS ONE Editorial Office Staff

on behalf of

Dr. Mohamed O Ahmed

Academic Editor

PLOS ONE
